# Cytotoxic Alkaloids Derived from Marine Sponges: A Comprehensive Review

**DOI:** 10.3390/biom11020258

**Published:** 2021-02-10

**Authors:** Ahmed M. Elissawy, Ebrahim Soleiman Dehkordi, Negin Mehdinezhad, Mohamed L. Ashour, Pardis Mohammadi Pour

**Affiliations:** 1 Department of Pharmacognosy, Faculty of Pharmacy, Ain Shams University, Cairo 11566, Egypt; aelissawy@pharma.asu.edu.eg (A.M.E.); ashour@pharma.asu.edu.eg (M.L.A.); 2 Medical Plant Research Center, Basic Health Sciences Institute, Shahrekord University of Medical Science, Shahrekord 88157-13471, Iran; amindehkordi78@gmail.com; 3 Department of Pharmacognosy, School of Pharmacy and Pharmaceutical Sciences, Isfahan University of Medical Sciences, Isfahan 81746-73461, Iran; negin.mehdinezhad@yahoo.com

**Keywords:** cytotoxicity, alkaloids, sponges, marine drugs, secondary metabolites

## Abstract

Marine sponges (porifera) have proved to be a prolific source of unique bioactive secondary metabolites, among which the alkaloids occupy a special place in terms of unprecedented structures and outstanding biological activities. Identification of active cytotoxic alkaloids extracted from marine animals, particularly sponges, is an important strive, due to lack of knowledge on traditional experiential and ethnopharmacology investigations. In this report, a comprehensive survey of demospongian bioactive alkaloids in the range 1987–2020 had been performed with a special emphasis on the potent cytotoxic activity. Different resources and databases had been investigated, including Scifinder (database for the chemical literature) CAS (Chemical Abstract Service) search, web of science, Marin Lit (marine natural products research) database. More than 230 representatives of different classes of alkaloids had been reviewed and classified, different genera belonging to the phylum porifera had been shown to be a prolific source of alkaloidal molecules, including *Agelas* sp., *Suberea* sp., *Mycale* sp., *Haliclona* sp., *Epipolasis* sp., *Monanchora* sp., *Crambe* sp., *Reniera* sp., and *Xestospongia* sp., among others. The sufficient production of alkaloids derived from sponges is a prosperous approach that requires more attention in future studies to consider the constraints regarding the supply of drugs, attained from marine organisms.

## 1. Introduction

Marine alkaloids present unique chemical structures that have been widely distributed among marine organisms. Some of them represent derivative molecules of the commonly encountered terrestrial alkaloids, whereas others show unprecedented novel structures confined to the marine systems. Their purification, structure elucidation, stereochemistry, chemical modification, synthesis, and pharmaceutical activity have acquired outstanding interdisciplinary attention from various fields of research aside from chemistry, including physiology, and pharmacology, ecology, biotechnology [1]. Alkaloids represent one of the most important classes of natural products that are widely distributed among different biological sources, including plants, animals, fungi, cyanobacteria, actinomycetes, dinoflagellates, red algae, cnidarians, and bryozoans; however, their presence in marine invertebrates as major constituents is limited to specific phylum, including some sponge genera, ascidians, mollusks, red algae, and bryozoans. Although the exact physiological function of alkaloids remains unclear, many of them had been developed as defense chemical weapons against predation—this is of great importance, especially for vulnerable sessile organisms, like sponges, and consequently, they are expected to be very potent molecules demonstrating toxicity at low doses [2,3,4].

This class of compounds demonstrates potent biological activities that can be considered as lead compounds for the development of potent antibiotics, antifungal, antiviral, anti-inflammatory, antimalarial, immune-modulating, or neuro-suppressive [5,6,7,8,9,10,11,12]. They also demonstrated promising cytotoxic activity versus diverse types of cancer cells [13,14]. Sponges (porifera), phylogenetically the oldest metazoan, have been recognized as a precious origin of unique secondary metabolites. About 5000 sponge species had been identified and classified with major groups belonging to Demospongiae [15]. They demonstrated wide distribution from intertidal coastal regions to great depths up to 8000 m depth [16]. The outstanding secondary metabolites produced by sponges are assumed to be the result of a combination of various factors, including the sessile nature of the organism, the porous nature of the body, and the environmental and ecological factors surrounding the organism [17]. As a consequence, it is obvious that these indefensible immobile organisms were provided by potent allelopathic factors that lead to the biosynthesis of many new drugs.

In this review, a comprehensive survey of about 240 alkaloids isolated from marine sponges from 1987 to 2020 had been performed, to shed light on those alkaloids demonstrating potent in vitro cytotoxicity, different resources, and databases, including Scifinder (database for the chemical literature) CAS (Chemical Abstract Service) search, web of science, Marin Lit (marine natural products research) database, were used. Moreover, review articles reviewing secondary metabolites from sponges were included [1,18,19,20,21,22]. Throughout this review, the alkaloids were classified into 20 different chemical classes (acridine, β-carboline, bromotyrosine, brominated, dimeric aaptamine, guanidine, imidazole, indole, peptide, piperidine, pyrimidine, pyridine, pyrrole, pyrroloiminoquinone, quinoline and quinolizidine, tetrahydroisoqouinoline, steroidal, terpenoidal, manzamine, and sesquiterpene quinones/hydroquinones alkaloid), and representative examples of each class have been reviewed with about 240 molecules described. It is worth noting that an individual alkaloid may fall in more than one chemical class, and placing certain alkaloid in a specific class was based on the authors’ classification in the original articles.

## 2. Alkaloid Classification

### 2.1. Acridine Alkaloids

**Figure 1:**
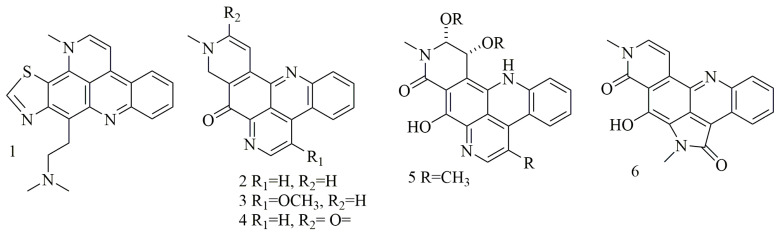
Structures of acridine alkaloids isolated from sponges.

Acridine nucleus (C_13_H_9_N) is a polycyclic heteroarene in which one of the central CH groups is replaced by a nitrogen atom. Chemical investigation of the *Dercitus* sp., collected from the Bahamas at 160 depth, led to the isolation of a violet pigment, identified as dercitin (**1**); cytotoxic evaluation of this compound against a panel of cell lines revealed potent cytotoxic activity in nanomolar concentration [23]. Investigation of different sponge samples belonging to genus *Xestospongia* collected from Indonesia, and New Guinea had led to the isolation of neoamphimedine (**2**), 5-methoxyneoamphimedine (**3**), amphimedine (**4**), neoamphimedine Z (**5**), alpkinidine (**6**). Compounds **2–6** were identified as bisannulated acridines. Based on results, compounds **2,3**, and **6** showed selective activity for solid tumors—among them, **2** was the most potent, and **3** showed the most selectivity for solid tumors [24]. Figure 1 illustrates the chemical structures of compounds **1–6**; Table 1 summarizes the cytotoxic evaluation of compounds **1–6**.

### 2.2. β-Carboline Alkaloid

**Figure 2:**
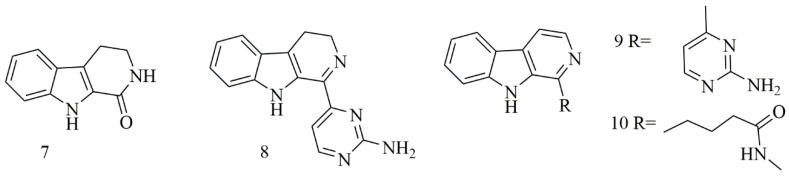
Structures of β-carboline alkaloids isolated from sponges.

Four β-carboline alkaloids 1,2,3,4-tetrahydronorharman-1-one (**7**) acanthomine A (**8**), annomontine (**9**), and ingenine E (**10**), were isolated from *Acanthostrongylophora ingens*. Compounds **7–10** showed the most potent cytotoxicity [25]. Figure 2 iIllustrates the chemical structures of compounds **7–10**; Table 2 summarizes the cytotoxic evaluation of compounds **7–10**.

### 2.3. Bromotyrosine Alkaloids

Qun Göthel et al. reported three bromotyrosine alkaloids identified as 14-debromo-11-deoxyfistularin-3 (**11**), aplysinin A (**12**), and aplysinin B (**13**) from the sponge *Aplysina lacunosa* collected from Stirrup Cay in the Bahamas. Compounds **11–12** revealed a unique, five-membered oxazole ring with a spiro atom linked with the bromocyclohexa-diene ring [26]. Investigation of different sponge samples belonging to genus *Hexadella* sp., *Jaspis* sp. and *Bubaris* sp., collected from Indonesia by Tarazona, and co-workers led to the isolation and identification of new bromotyrosine alkaloids **14–15**. Aplyzanzine B (**14**) was isolated from *Jaspis* sp., and *Bubaris* sp., whereas Anomoian B (**15**) was isolated from *Hexadella* sp. have shown potent cytotoxic activity against three human tumor cell lines A549, HT-29, and MDA-MB-231 (6.1, 1.6, and 7.8 μM, respectively) [27].

**Figure 3:**
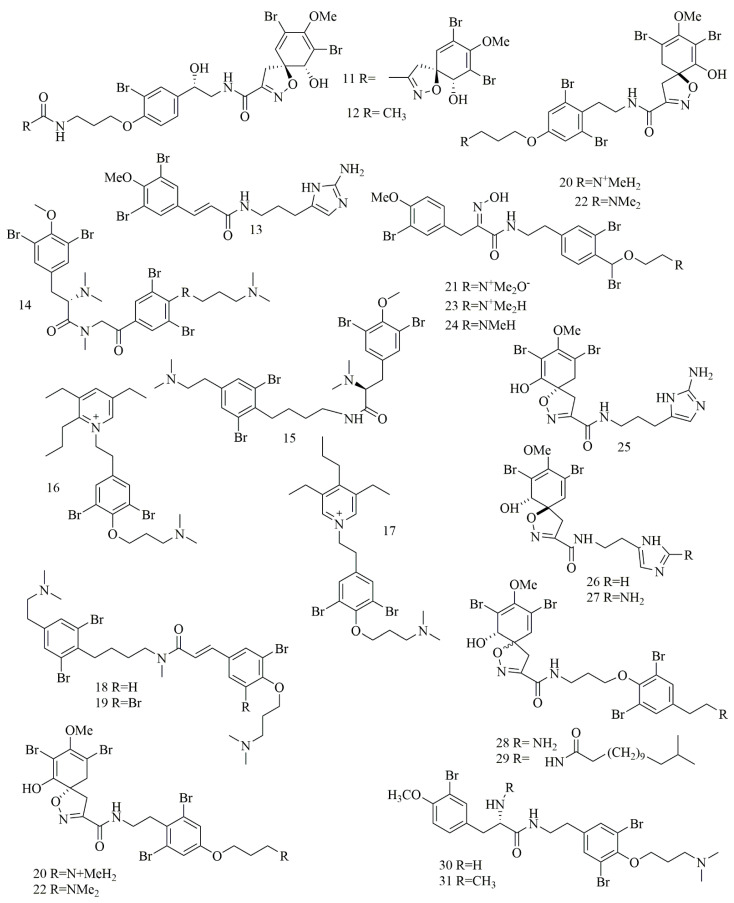
Structures of bromotyrosine alkaloids isolated from sponges.

In another study, ma’edamines C (**16**) and ma’edamines D (**17**) were isolated by Japanese researchers from the Okinawan marine sponge *Suberea* sp. compounds **16–17** revealed cyclization of the side-chain nitrogen of tyrosine to add a quaternary pyridinium nucleus to the structure. Both compounds have shown selective cytotoxicity against murine leukemia L1210 cell line though, and they did not express cytotoxicity against KB cell line [28]. Investigation of the marine sponge *Psammoclemma* sp. collected from Bommie Bay, Queensland, Australia, led to the isolation of psammaplysene C (**18**) and psammaplysene D (**19**). Compounds **18–19** showed C6C3N moiety in one of their bromotyrosine uints instead of the conventional C6C2N arrangement [29]; both compounds showed potent cytotoxic activities. Another bromotyrosyn alkaloids were isolated from the Fijian sponge *Druinella* sp. These 10 bromotyrosine alkaloids **20–29**, were identified as purealidin S (**20**), purpuramine J (**21**). PurealidinQ (**22**), aplysamine 2 (**23**), purpureamine I (**24**), aerophobin2 (**25**), aerophobin1 (**26**), purealidin J (**27**), araplysillin1 (**28**), and araplysillin 2 (**29**). Compounds **25–27** revealed replacing the other bromobenzene moiety commonly encountered in bromotyrosine alkaloids with an imidazole ring. Compounds **20–29** were evaluated for their cytotoxicity against two cell lines showing potent to moderate activities. Among them, compound **21** was the most potent cytotoxic compound [30]. Suberedamine A (**30**) and suberedamine B (**31**) were isolated from *Suberea* sp. These two new cytotoxic bromotyrosine alkaloids exhibited potent cytotoxic activity against L1210 and KB cell lines [31]. Figure 3 illustrates the chemical structures of compounds **11–31**; Table 3 summarizes the cytotoxic evaluation of compounds **11–31**.

### 2.4. Dibrominated and Brominated Alkaloids

**Figure 4:**
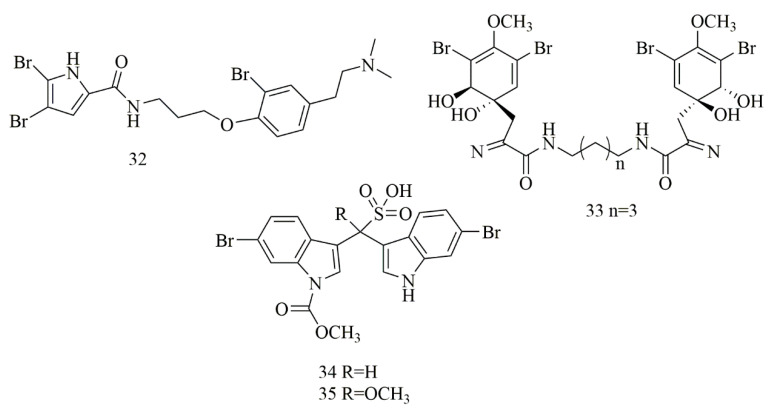
Dibrominated and brominated alkaloids from marine sponges.

Tilvi et al. reported three new pyrrole-2-aminoimidazole alkaloids from the *Agelas dendromorpha* collected from New Caledonia. Cytotoxic investigation of the isolated compounds revealed that only one compound identified as Agelastatin E (**32**) that showed 100% activity at 3 and 30 µM against KB cell lines [32]. In Shaala et al. a dibrominated alkaloid, aerothionin (**33**) with potent cytotoxicity against HeLa cells was identified from the sponge, *Suberea* sp., collected from the Red Sea in Yanbu, Saudi Arabia [33]. Two brominated indolosulfonic acid derivatives were reported from the hydroalcoholic extract of the *Psammoclemma* sp., collected from New Caledonia. The compounds were identified as echinosulfonic acid D (**34**) and echinosulfonic acid B (**35**) based on extensive LC/MS/MS analysis besides conventional 1 and 2 D NMR analysis. Both compounds showed potent cytotoxicity against KB cells with equal IC_50_ of 2 µg/mL [34]. Figure 4 illustrates the chemical structures of compounds **32–35**; Table 4 summarizes the cytotoxic evaluation of compounds **32–35**.

### 2.5. Aaptamine Alkaloids

**Figure 5:**
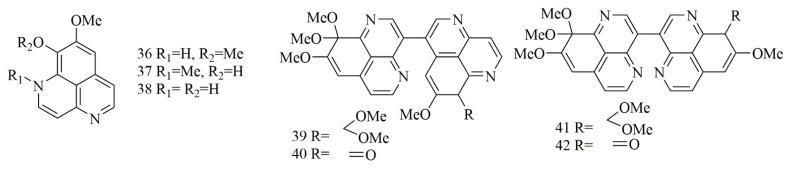
Dimeric aaptamine alkaloids from marine sponges.

Bioassay-guided fractionation of the Indonesian sponge *Aaptos suberitoides* resulted in the isolation of three benzonaphthryidine derivatives identified as aaptamine (**36**), isoaaptamine (**37**), and demethylaaptamine (**38**). Biological evaluation of compounds **36–38** revealed potent cytotoxicity against HeLa cell lines with IC_50_ values of 15, 3.1, and 1.4 µg/mL, respectively. Moreover, the activity had been attributed to proteasome inhibitory action [35]. Investigation of a different sample from the same sponge *A. suberitoides*, collected from Xisha islands in the South China Sea (NaiHai), has led to the isolation of additional aaptamine derivatives with dimerization in the benzonathyridine moiety, the compounds were identified as suberitine A (**39**), suberitine B (**40**), suberitine C (**41**) and suberitine D (**42**). Cytotoxic evaluation of **39–42** against P388, HeLa, and K562 cell lines revealed selective, potent activity of compounds **40** and **42** with IC_50_ values of 1.8 and 3.5 μM, respectively, against P388 cell line with the absence of activity on other lines. Interestingly, compounds **39** and **41** did not show significant effects against any of the cell lines, revealing the possible need of exocyclic double bond in one of the monomer units either as ketone as in **42** or exocyclic terminal methylene as in **40** [36]. Figure 5 illustrates the chemical structures of compounds **36–42**; Table 5 summarizes the cytotoxic evaluation of compounds **36–42**.

### 2.6. Guanidine Alkaloids

**Figure 6:**
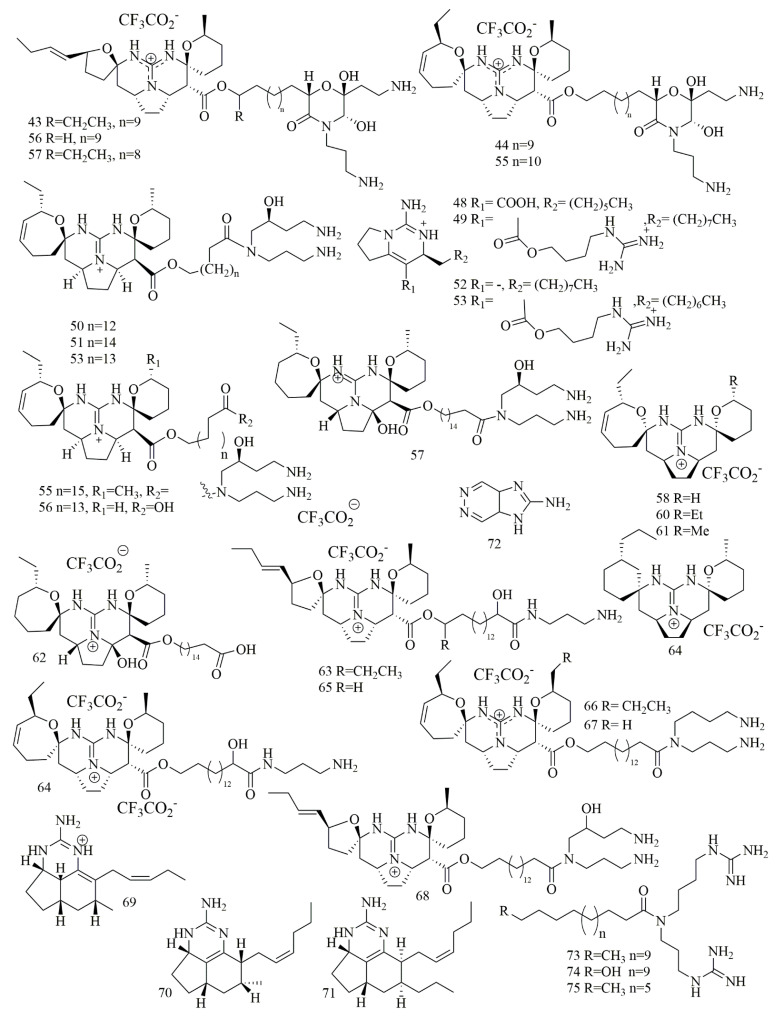
Guanidine alkaloids from marine sponges.

*Monanchora pulchra* collected from the Pacific Ocean near Urup islands, was the source of guanidine alkaloid monanchocidin A (**43**). It is composed of a five-membered spiro-ring in the pentacyclic guanidine core along with an uncommon branched long alkyl chain and a heavily oxygenated morpholinone fragment [37]. Evaluation of **43** against monocytic anemia cell lines THP-1, Hela, and JB6-C141 cell lines revealed potent cytotoxicity with IC_50_ values against 5.1 µM, 11.8 µM, and 12.3 µM, respectively [38]. As this sponge is a great source of guanidine alkaloids, additional pentacyclic guanidine alkaloids, monanchocidin B-E (**44–47**) were reported by Makarieva at al. Compounds **44–47** revealed strong inhibitory activities with IC_50_ values of 540, 200, 110, 830, and 650 nM, respectively against HL-60 cells [39]. Investigation of different sample of the *Monanchora* sp. sponge, collected from a depth of 20 m from the coast of Hiva Oa Island (French Polynesia) led to the isolation of nine cytotoxic pentacyclic guanidine alkaloids identified as monanchoradin A (**48**), dehydrocrambescin A2 418(**49**), crambescidin 786(**50**), (−)-crambescidin 814 (**51**), monalidine A (**52**), (−)-crambescin 406 (**53**), crambescidin 800 (**54**), crambescidin 826 (**55**) and 20-norcrambescidic acid (**56**). Cytotoxic evaluation of compounds **48–56** had been performed against a panel of cell lines revealed interesting cytotoxic activity [40]. Crambescidin-816 (**57**) has been isolated from *Crambe crambe* (oyster sponge) belongs to the pentacyclic guanidine alkaloid. Biological evaluation of **57** revealed its ability to reduce cell viability of the tumor-derived cell line HepG2 at concentrations higher than 150 nM. Compound **57** also affected the human tumor-derived cell lines OVCAR (ovary carcinoma), HOP-92 (pulmonary carcinoma), MCF-7, PC3 (prostatic carcinoma), SK-MEL-28 (melanoma), UO-31 (kidney carcinoma), and HT-29 by reducing the cell viability at the three concentrations in all the cell lines except MCF-7 [41]. Detailed biological study of **57** attributed to its activity on calcium voltage-dependent channel blockage and decreasing neuronal viability in cortical neurons [42]. Extract of the Marine sponge *Clathria bulbotoxa*, collected from Samalona island, Indonesia, yielded five cytotoxic guanidine alkaloids **58–62**, identified as crambescidins 345 (**58**), crambescidins 361 (**59**) crambescidins 373 (**60**), crambescidins 359 (**61**), and crambescidins 657 (**62**). All compounds demonstrated cytotoxic activity less than 10 μM against A431 cell line. Compounds **61** and **62** had the most powerful cytotoxicity with IC_50_ of 12 and 48 nM. Also **58–60** had modest cytotoxic with IC_50_ of 7.0, 2.5, 0.94 and 3.1 μM [43]. Continued investigation of the sponge *Monanchora pulchra* had has yielded three pentacyclic guanidine alkaloids normonanchocidin A (**63**), normonanchocidins B (**64**), and normonanchocidin D (**65**). Compounds **64** and **65** were not isolated in pure form and usually yielded a 1:1 mixture. The cytotoxic activity of compounds **63** and the mixture of **64** and **65** (1:1) against THP-1 showed IC_50_ values of 2.1 µM, 3.7 µM, 3.8 µM, and 6.8 µM, against HeLa cells, respectively [44]. Additionally, two pentacyclic guanidine alkaloid, namely, monanchomycalin C (**66**) and ptilomycalin A (**67**), were found in a different sample of *Monanchora pulchra* (Lambe, 1894, family Crambeidae) collected from Kunashir Island. The cytotoxic activity of the **66** and **67** against human breast cancer MDA-MB-231 cells displayed IC_50_ values of 8.2 µM and 4.3 µM, respectively [45]. Another different sample of *Monanchora pulchra* collected off the Chirpoi sland was a source of monanchoxymycalin C (**68**), a pentacyclic guanidine alkaloid. Compound **68** was able to inhibit the colony formation of HeLa cells, at a concentrations of ≥1.25 μM [46]. Tricyclic guanidine alkaloids have been reported from some sponges, including *Biemna laboutei*, collected from Salary Bay, Madagascar, seven Tricyclic guanidine alkaloids, were isolated from the methylene chloride: Methanol extract of this sponge, however only one compound, netamine M (**69**), revealed potent cytotoxicity against KB cell line with IC_50_ value of 1 μM [47]. Further investigation on *B. laboutei*, led to the isolation of additional seven netamine derivatives, among which, only netamine O (**70**), netamine Q (**71**), revealed cytotoxic activity against KB cells in the range of 10 µM [48]. Zanissine (**72**), a new bicyclic 4, 5-guanidino-pyridaine alkaloid was yielded from the CH_2_Cl_2_ extract of *Anchinoe pauperta*, collected from Zarzis, Tunisia. Compound **72** showed cytotoxic activity against P-388, KB, and NSCLC-N6 cell lines with IC_50_ of 12 μg/mL, 5 μg/mL, and 10 μg/mL [49]. The Marine sponge *Monanchora* sp., collected off the coast of Hiva Oa Island (French Polynesia) at 20 m depth, yielded three acyclic cytotoxic bis-guanidine alkaloids Unguiculin A (**73**), Unguiculin B (**74**), and Unguiculin C (**75**). Cytotoxic evaluation of **73–75** against KB cancer cell lines had revealed potent cytotoxicity with IC_50_ of 0.2, 0.08, and 0.03 μM, respectively [50]. Figure 6 illustrates the chemical structures of compounds **43–75**; Table 6 summarizes the cytotoxic evaluation of compounds **43–75**.

### 2.7. Imidazole Alkaloid

**Figure 7:**
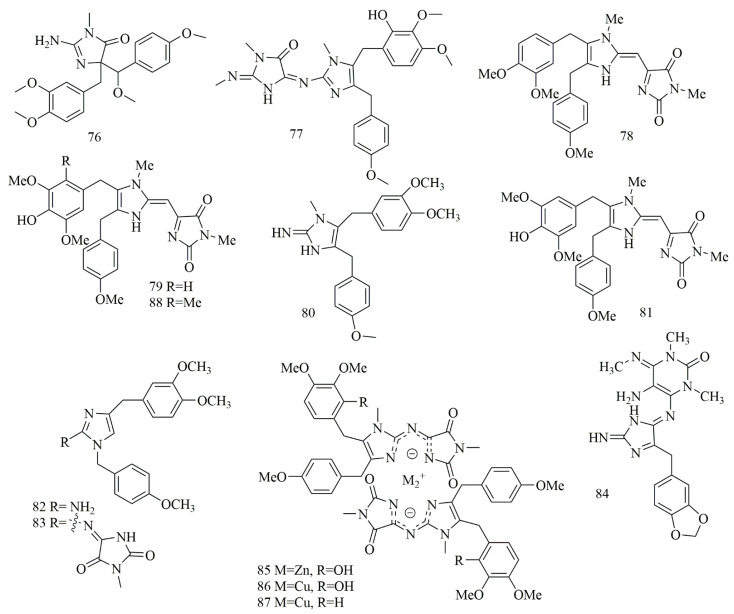
Cytotoxic imidazole alkaloid structures in marine sponges.

Imidazole metabolites were reported from the Chinese sponge *Leucetta chagosensis* collected off the Yongxing islands in the South China Sea and yielded (−)-calcaridine (**76**) and (2E, 9E)-pyronaamidine-9-(N-methylimine) (**77**). Both compounds revealed selective cytotoxicity against MCF-7 cell lines with IC_50_ values of 25.3 and 24.2 μM, respectively, but no cytotoxic effect was detected on A549 and PC9 cell lines [51]. Two cytotoxic bi-imidazole alkaloids, naamidine J (**78**) and naamidine H (**79**) were isolated from *Pericharax heteroraphis* collected from the South Sea (Yongxing islands) at a depth of 12 m. Cytotoxic evaluation of the two alkaloids **78–79** revealed potent activity against cancer cell lines, where compound **78** inhibited the growth of K562 cell line with IC_50_ values of 11.3 µM, on the other hand, compound **79** showed an inhibitory effect on K562, HeLa, and A549 cell lines with IC_50_ values of 9.4, 21.4 and 22.4 µM, respectively [52]. Naamine J (**80**), a guanidine-based imidazole alkaloid was isolated from *Leucandra* sp., collected off Woody islands in the South China Sea. Evaluation of the cytotoxic effects of **80** on four MCF-7, A549, HeLa, and PC9 cell lines showed it possessed an inhibitory effect with IC_50_ values of 20.1, 23.7, 28.2, and 45.3 μM, respectively [53]. Naamidine I (**81**) along with **79** were isolated from *Leucetta chagosensis* (Calcarea sponge) collected from a depth of ten meters in North Sulawesi, Indonesia. Compounds **79** and **81** revealed cytotoxic effect on the HeLa cell line with IC_50_ values of 11.3 and 29.6 μM, respectively [54]. Isonaamine C (**82**), isonaamidine E (**83**), and two cytotoxic imidazole alkaloids isolated from *Leucetta chagosensis* from Bougainville Reef, the Great Barrier Reef, Australia. Cytotoxic effects of all compounds on HM02, HepG2, and Huh7 cell lines showed that compound **83** had growth inhibitory effect with GI_50_ values of 15.1, 15.1, and 2.8 µM, respectively. Whereas, compound **82** showed GI_50_ values of 15.0, 6.2, and 5.9 µM, respectively [55]. Another imidazole alkaloid, leucosolenamine B (**84**), isolated from *Leucosolenia* sp., collected off Milne Bay in Papua New Guinea, showed potent cytotoxic effect on the C-38 cell line with IC_50_ value of 19.6 µM [56]. Further investigation demonstrated *Leucetta chagosensis* collected from the South China Sea is a source of unique metal complexes, chagosendine A (**85**), chagosendine B (**86**), and chagosendine C (**87**) along with pyronaamidine (**88**) obtained by reacting chagosendine B with Na_2_S and **80** from reacted chagosendine C with Na_2_S. Cytotoxic evaluation of **86** and **87** showed potent inhibition against K562, HepG2, and HeLa cell lines, where **86** inhibited K562, HepG2, and Hella with IC_50_ of 0.62, 1.19, and 0.58 μM and **87** showed IC_50_ value of 0.62, 0.31 and 4.43 μM, respectively. Whereas, **85** displayed weak activity IC_50_ more than 10 μM against the cell lines. Compound **88** inhibited K562 and Hela with IC_50_ values of 6.87 and 5.62 μM [57]. Figure 7 illustrates the chemical structures of **76–88**; Table 7 summarizes the cytotoxic evaluation of **76–88**.

### 2.8. Indole, Bisindole, and Trisindole Alkaloids

**Figure 8:**
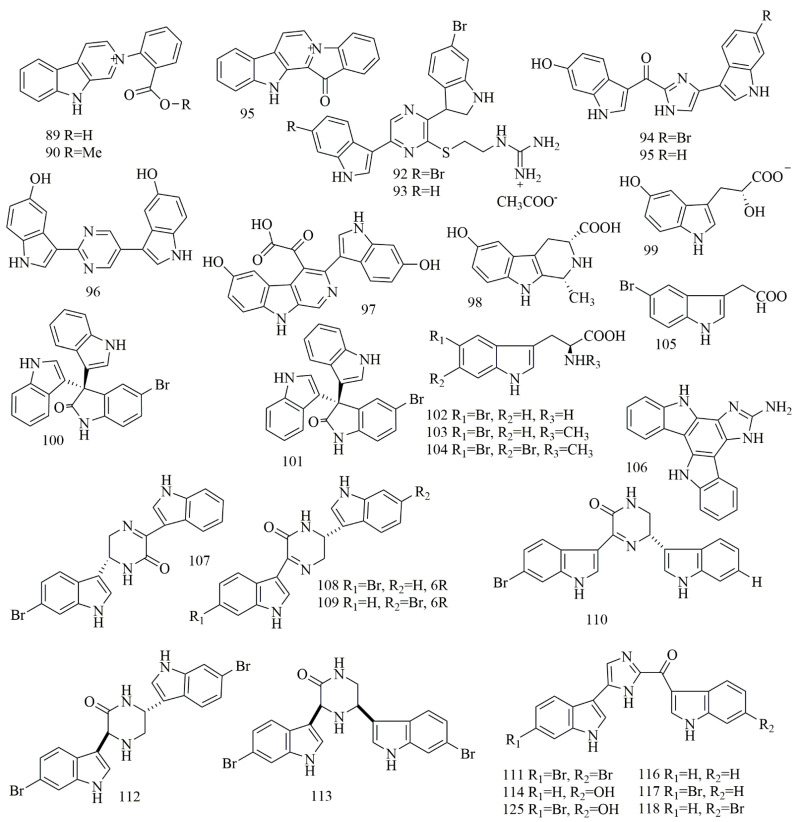
Cytotoxic Indol, Bisindol, and Trisindol alkaloid structures in marine sponges.

The aqueous extract of the sponge *Thorectandra* sp., was a source of quaternary indole alkaloids identified as demethoxyfascaplysin (**89**), 1-deoxysecofascaplysin A (**90**), and fascaplysin (**91**). Compound **89** showed cytotoxic effects on breast cancer cell line (IC_50_ of 20.4 µM), **90** inhibited the growth of MCF-7, OVCAR-3, and A549 (IC_50_ of 4.9, 7.2 and 43.2 µM), whereas compound **91** showed cytotoxicity (IC_50_ 0.11–1.40 µM) against MCF-7, OVCAR-3, MALME-3M and A549 [58]. Four bisindole alkaloids identified as dragmacidin G (**92**), dragmacidin H (**93**), topsentin B2 (bromotopsentin) (**94**), and topsentin B1 (topsentin) (**95**) were reported from *Lipastrotethya* sp., (Dictyonellidae family) collected off north of Hachijo Island, Japan. Compounds **92–95** exhibited cytotoxicity on HeLa cells with IC_50_ values of 4.2, 4.6, 1.7, and 4.4 μM, respectively [59]. LCMS analysis of a *Hyrtios* sp. extract, collected from Unten-Port, Okinawa, yielded 2,5-disubstituted pyrimidine bisindole alkaloid hyrtinadine A (**96**), with potent cytotoxicity on L1210 and KB cell lines with IC_50_ of 2.9 and 8.7 µM [60]. *Hyrtios erectus* collected from Hurghada, Egypt, was a source of indole alkaloids those were identified as hyrtioerectine A (**97**), hyrtioerectine B (**98**), and hyrtioerectine C (**99**). Compounds **97–99** demonstrated cytotoxic effects on HeLa cell line with IC_50_ (25.8, 20.3, and 20.4 μM) [61]. Two brominated tris-indole alkaloids were isolated from *Callyspongia siphonella*, collected from Hurghada, Egypt. The two alkaloids were identified as 5-bromotrisindoline (**100**) and 6-bromotrisindoline (**101**). Results from HT-29, OVCAR-3, and MM.1S cell line displayed that **100** and **101** proved to be toxic with IC_50_ values 8, 7, and 9 μM, for 100 and 12.5, 9, and 11 μM, for **101** [62]. Four simple bromoindole alkaloids, 5-bromo-l-tryptophan (**102**), 5-bromoabrine (**103**), 5,6-dibromoabrine (**104**), and 5-bromoindole-3-acetic acid (**105**), were isolated from *Smenospongia* sp., collected from Batanes, Philippines. Compounds **102–105** were evaluated against HCT-116 cell lines by means of an MTT assay, and they exhibited the least cytotoxicity in a set of isogenic HCT116 cell lines. Based upon the result the reduction in activity against the p53−/− cell line was evidenced [63]. Damirine A (**106**), a guanidine-based bis-indole alkaloid, was isolated and identified from the sponge *Damiria* sp., collected from Phuket Island, Thailand. Results from MALME-3M, Sw620, HCC-2998, MOLT-4, k562cell line revealed that **106** proved to be cytotoxic with GI_50_ of 1.9, 3.3, 2.3, 1.9, 2.2 μM [64]. 6″-Debromohamacanthin A (DBHA) (**107**), another bisindole alkaloid, was identified in a few sponges, including *Spongosorites* sp., and **107** did not show substantial cell viability up to 10 μM, but over 20 μM DBHA displayed a cytotoxic effect in mES cell line (IC_50_: 28.5 μM) [65]. Biossay-guided isolation of the methanol extract of *Spongosorites* sp., collected from the coast of Jeju Island, Korea, yielded the isolation of indole alkaloids (**108–118**), identified as I-6′′-debromohamacanthin A (**108**), (R)-6′-debromohamacanthin A (**109**), and (S)-6′′-debromohamacanthin B (**110**), categorized as hamacanthin class and dibromodeoxytopsentin (**111**) a bisindole alkaloid of the Topsentin class, along with trans-3,4-dihydrohamacanthin A (**112**), cis-3,4-dihydrohamacanthin B (**113**), topsentin(**114**), bromotopsentin (**115**), deoxytopsentin (**116**), bromodeoxytopsentin (**117**) and isobromodeoxytopsentin (**118**). Compounds **108–118** were evaluated for cytotoxicity against A549, SK-OV-3, SK-MEL-2, XF498, and HCT15 cell lines. Compounds **108**, **112**, **113**, and **118** displayed modest to substantial cytotoxicity to all of the cancer cell lines established. Compounds **114** and **115** displayed cytotoxicity on P388 with IC_50_ values of 5.8 and 16.6 µM, respectively. Compounds **117** and **118** were described to exhibit cytotoxicity against the cell-line K-562 with IC_50_ values of 1.4 and 5.1 µM, respectively [66]. Figure 8 illustrates the chemical structures of compounds **89–118**; Table 8 summarizes the cytotoxic evaluation of compounds **89–118**.

### 2.9. Peptide Alkaloid

**Figure 9:**
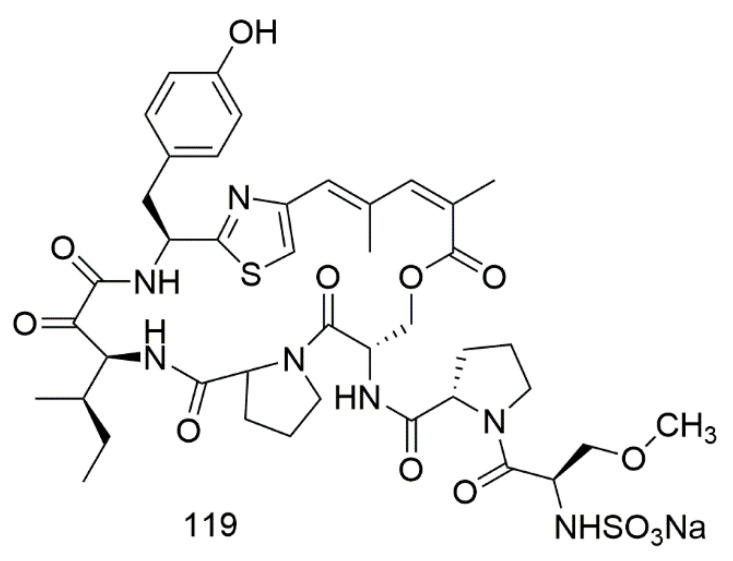
Peptide alkaloids from sponge.

Scleritodermin A (**119**), a potent sulfonated thiazol hexapeptide was isolated from the marine sponge *Scleritoderma nodosum* collected from the northwest side of Olango Island, Cebu, Philippines. The purified compound demonstrated low µM cytotoxicity to HCT116, A2780, and SKBR3 cell lines with IC_50_ of 1.9, 0.940, and 0.670 µM, respectively [67]. Figure 9 illustrates the chemical structures of compound **119**; Table 9 summarizes the cytotoxic evaluation of compound **119**.

### 2.10. Piperidine Alkaloids

**Figure 10:**
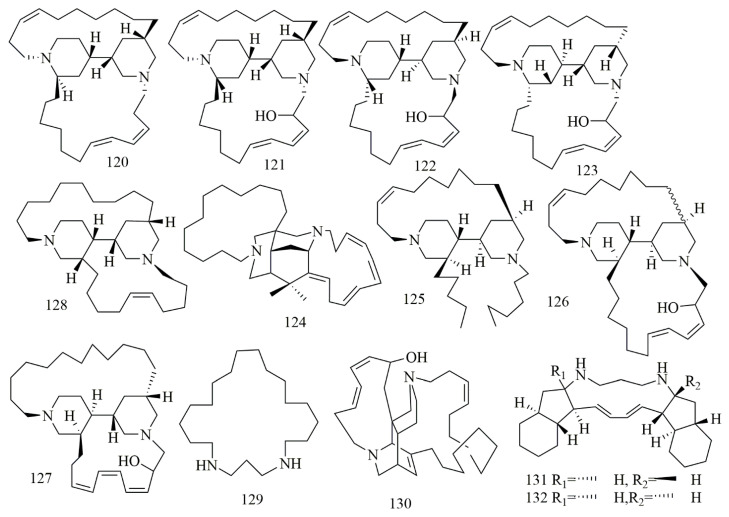
Piperidine alkaloid structure in marine sponges.

*Arenosclera brasiliensis* endemic to the southeastern Brazilian coastline collected from Joa˜o Fernandinho Beach, Bu’zios (Rio de Janeiro state) was the source of alkylpiperidine alkaloids identified as arenosclerin A (**120**), arenosclerin B (**121**), arenosclerin C (**122**) and haliclonacyclamine E (**123**). Results of cytotoxic activity displayed that **121–123** had cytotoxic effects in the range of 0.5–2.1 μg/mL to HL-60, B16, L929, and U138 cancer cell lines [68]. Madangamine F (**124**) haliclonacyclamine F (**125**), arenosclerins D (**126**), and arenosclerins E (**127**), were found in *Pachychalina alcaloidifera* as one of the richest national source of bis-pyridine alkaloid. Results proved the cytotoxic activity of **124–127** on SF295, MDA-MB435, HCT8, and HL60 cell lines. The most potent activity was against MDA-MB435 cell lines with IC_50_ values 16.2, 1, 1.2, and 3.1 μg/mL, respectively [69]. Neopetrosiamine A (**128**) was isolated from *Neopetrosia proxima* collected from Mona Island of Puerto Rico, which is belonging to the order Haplosclerida that is an abundant source of 3-alkylpiperidine alkaloids. Compound **128** displayed inhibitory effect on MALME-3M, CCRF-CEM, and MCF7 with IC_50_ values of 1.5, 2.0, and 3.5 μM, respectively [70]. Further, 1,5-diazacyclohenicosane (**36**) isolated from the marine sponge *Mycale* sp., collected from Kenya, was identified as a new macrocyclic diamine alkaloid. The compound showed interesting cytotoxic activity against the panel of cell lines [71]. Ingenamine G (**129**), isolated from *Pachychalina* sp., collected in Ilha do Pai (Father’s Island), NiteIo´I, Rio de Janeiro. Compound **129** was identified as macrocyclic dipipridine alkaloid. Cytotoxic evaluation of **129** against HCT-8, B16, and MCF-7 revealed potent cytotoxicity [72]. Papuamine (**130**) and haliclonadiamine (**131**) were identified as polycyclic alkaloids isolated from *Neopetrosia cf exigua* collected from Old Derawan Pier, Indonesia, at depth of 24 m. Compounds **130** and **131** were tested on human glioblastoma cell line (SF-295), and human renal cancer cell lines (UO-31 and A498)—and results proved their cytotoxic effects; in another study, **130** was reidentified from *Haliclona* sp, also collected from Indonesia, with remarkable cytotoxicity on MCF-7 cell [73,74]. Figure 10 illustrates the chemical structures of **120–131**; Table 10 summarizes the cytotoxic evaluation of **120–131**.

### 2.11. Pyrimidine Alkaloids

**Figure 11:**
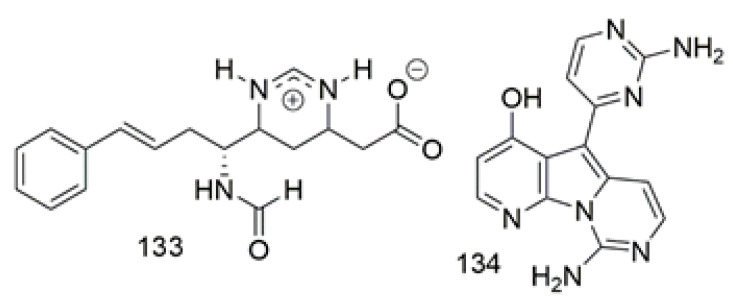
Pyrimidine alkaloid structure isolated from sponges.

Lanesoic acid (**133**), was isolated as zwitterionic alkaloid from *Theonella swinhoei* a sponge of Theonella family collected from Lanes in Indo-Pacific, Indonesia. Compound **133** displayed an unusual 1, 4, 5, 6-tetrahydropyrimidine cation rarely exhibits in natural sources. Compound **133** was evaluated for cytotoxicity against a panel of cell lines, such as PSN1, HT-29, breast MD-MB-231, and A549, that revealed selective cytotoxicity against PSN1cells with an IC_50_ value of 28.2 μM and was inactive against NSCLC lung tumor, MD-MB-23, and HT-29 cells [75]. The alkaloid variolin B (**134**), heterocyclic constitute from the Antractic sponge *Kirkpatrickia variolosa* had been evaluated for cytotoxicity against a wide range of cancer cell lines, where it showed potent activity against most of the tested cell lines with the activity against androgen-sensitive prostate adenocarcinoma cell line with GI_50_ 0.05 µM [76]. Figure 11 illustrates the chemical structures of **133–134**; Table 11 summarizes the cytotoxic evaluation of **133–134**.

### 2.12. Pyridine Alkaloids

**Figure 12:**
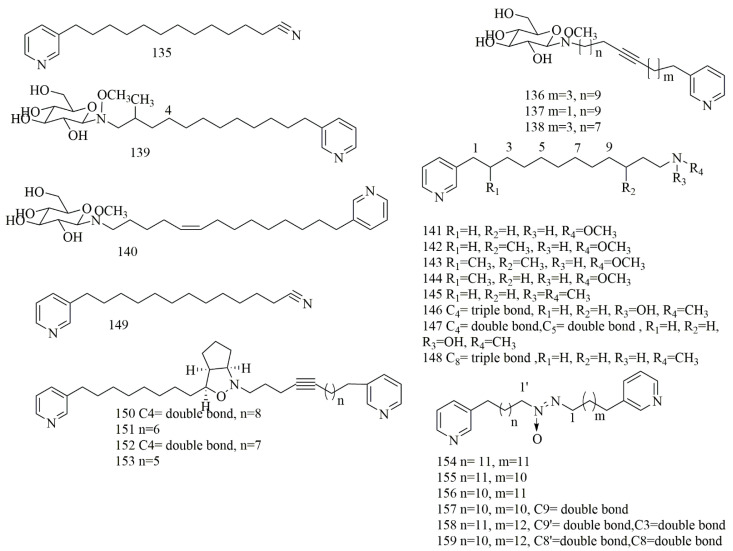
Pyridine alkaloids from sponges.

The sample of *Haliclona* sp., collected from Indonesia, contained pyridine alkaloid cyano -3-dodecyl pyridine (**135**). Compound **135** showed moderate cytotoxity against A549, MCF-7, and Hela cell lines with IC_50_ values of 41.8, 48.4, and 33.2 μM, respectively [77]. Four pyridine glycosides had been isolated from *Amphimedon* sp., collected from Hachijo Island, Japan. The glycosidal alkaloids were identified as amphimedoside A (**136**) as a β-glucopyranose, amphimedoside C (**137**), a positional isomer of amphimedoside B (**138**) in the acetylenic bond with same molecular formula C_26_H_42_N_2_O_6_, amphimedoside D (**139**) and amphimedoside E (**140**). The results proved the cytotoxicity of **136–140** against P388 with IC_50_ values 90.0, 10.4, 23.0, 0.9, and 4.5 µM, respectively. These β-glucopyranosyl 3-alkylpyridine alkaloids exhibited comparable cytotoxic activities with aglycones [78]. Interestingly, from two different sponges *Xestospongia* sp. and *Amphimedon* sp., collected from Hachijo island, Japan l seven related 3-alkylpyridine alkaloids were reported: Hachijodine A (**141**) hachijodine B(**142**), hachijodine C(**143**), and hachijodine D(**144**) from *Xestospongia* sp., and hachijodine E (**145**), hachijodine F (**146**), and hachijodine G (**147**) from *Amphimedon* sp. [79]. N-methylniphatyne A (**148**), a 3-alkylpyridine alkaloid, was isolated from marine sponge of *Xestospongia* sp., collected off Java Island, Indonesia. Compound **148** was identified as N-methyl derivative of niphatyne A (**149**), which was reported from Fijian sponge of *Niphates* sp. Compound **148** revealed potent cytotoxicity against PANC-1 cells with IC_50_ value of 16 µM, whereas **149** showed cytotoxic effect of 2 µM on P388 cells [80]. Pyrinodemin A-D (**150–153**) were isolated from Okinawan sponge *Amphimedon* sp., collected off Nakijin, Okinawa. Compounds **151–153** showed strong cytotoxicity on L1210 with IC_50_ value (0.12, 0.10 and 0.14 µM, respectively) and KB with IC_50_ value (0.89, 0.89, 0.91 µM, respectively) [81]. Pyrinadines B-G (**154–159**), bis-3-alkylpyridine alkaloids with an azoxy moiety, were reported from the Okinawan sponge *Cribrochalina* sp., collected from Unten Port, Okinawa. Compounds **154–159** showed potent cytotoxic effects on L1210 cell line with IC_50_ values (23.0, 18.1, 17.3, 16.0, 11.9 and 11.9 µM, respectively). However, they did not this effect on KB cell line (IC_50_ > 20 µg/mL) [82]. Figure 12 illustrates the chemical structures of **135–159**; Table 12 summarizes the cytotoxic evaluation of **135–159**.

### 2.13. Pyrrole and Bromopyrrole Alkaloids

**Figure 13:**
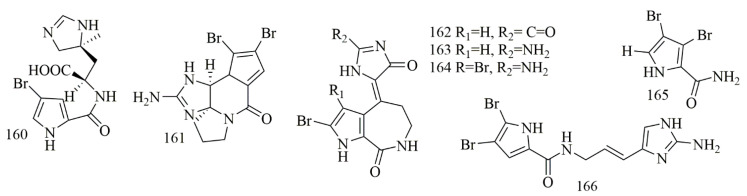
Pyrrole and bromopyrrole alkaloid structures from sponges.

*Stylissa carteri* collected from the Red Sea, Hurghada, Egypt, was the source of (−) clathramide C (**160**) (+)-dibromophakelline (**161**), (*Z*)-spongiacidin D (**162**), (*Z*)-hymenialdisine (**163**), (*Z*)-3-bromohymenialdisine (**164**) and 3,4-dibromo-1H-pyrrole-2-carbamide (**165**). Compounds **160–164** were evaluated for their cytotoxic activity against L5178Y and HCT116 cell lines. Regarding L5178Y cell line, **161** and **164** displayed cytotoxic effects with inhibition of growth 57.0% and 60.5%, respectively (10 μg/mL), while the cytotoxicity of **160**, **162**, **163** and **165** were not potent (growth inhibition of 25.3%, 36.7%, 37.0% and 38.4%, respectively) [83]. Oroidin (**166**), a pyrrole alkaloid from *Agelas oroides*., showed the cytotoxic activity with the GI_50_ of 42 µM on MCF-7, 24 µM on A2780 cells and more than 50 µM on HT29, SW480, H460, A431, Du145, BE2-C, SJ-G2, MIA, SMA, and U87 cell lines [84]. Figure 13 illustrates the chemical structures of **160–166**; Table 13 summarizes the cytotoxic evaluation of **160–166**.

### 2.14. Pyrroloiminoquinone Alkaloids

**Figure 14:**
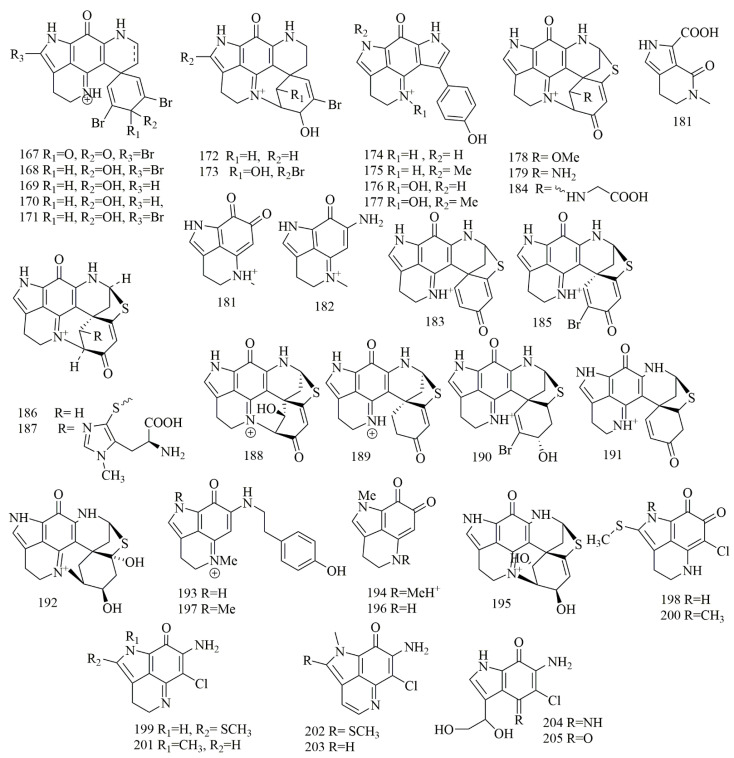
Pyrroloiminoquinone alkaloids structures isolated from sponges.

Four different species of marine sponges collected off Algoa bay of South Africa, namely, *Tsitsikamma pedunculata*, *Tsitsikamma favus*, *Latrunculia bellae*, *Strongylodesma algoaensis*, had been investigated for cytotoxic alkaloids yielding twenty one alkaloidal pigments (**167–187**) belong to pyrroloiminoquinone class and could be describesd as pyrroloacridine alkaloids, the compounds were identified as 14-bromodiscorhabdin C (**167**), 14-bromo-3-dihydrodiscorhabdin C (**168**), 3-dihydrodiscorhabdin C (**169**), 3-dihydro-7,8-dehydrodiscorhabdin C (**170**), 14-bromo-3-dihydro-7,8-dehydrodiscorhabdin C (**171**), discorhabdin V (**172**), and 14-bromo-1-hydroxydiscorhabdin V (**173**), from *T. pedunculata*. Tsitsikammamine A (**174**), tsitsikammamine B (**175**), tsitsikammamine A N-18 oxime (**176**), and tsitsikammamine B N-18 oxime (**177**), from *T.favus*. 1-Methoxydiscorhabdin D (**178**), 1-aminodiscorhabdin D (**179**), damirone B (**180**), makaluvic acid A (**181**), makaluvamine C (**182**), discorhabdin G (**183**), and discorhabdin N (**184**), from *L. bellae*. Discorhabdin A (**185**), discorhabdin D (**186**), discorhabdin H (**187**), from *S. algoaensis*. All the purified compounds **167–187** revealed potent cytotoxic activity against HCT-116 cell lines. Among them, **185** was the most potent with IC_50_ value of 0.007 µM, the tsitsikammamines intercalate DNA and cleave DNA via inhibition of topoisomerase I [85]. Further discorhabdins were reported from the Patagonian sponge *Latrunculia brevis*, collected from Patagonia, Argentina. These compounds identified by spectroscopic analysis as discorhabdin L (**188**), and discorhabdin I (**189**), showed potent cytotoxic effects on the HT-29 cell line with GI_50_ values of 0.12 and 0.35 μM, respectively [86]. Chemical investigation on *Higginsia* sp., collected from Deal island led to the purification of **185** and **186**. In addition, (+)-dihydrodiscorhabdin A (**190**), (+)-debromodiscorhabdin A (**191**), was obtained from *Spongosorites* sp., collected from Port Campbell along with (+)-discorhabdin X (**192**), makaluvamine J (**193**), damirone A (**194**), and (+)-dihydrodiscorhabdin L (**195**). Compounds **185**, **190**, **193–195** were subjected to cytotoxic assay against a panel of cell lines. Compounds **193–194** showed the least activity (IC_50_ 5–10 μg/mL). Compounds **190** and **195** showed more potent activity with IC_50_ 0.1–0.5 μg/mL, and finally, **185** showed the most potent cytotoxicity with IC_50_ 0.05–0.1 μg/mL [87]. Deniz Tasdemir et al. investigated the Philippine sponge *Smenospongia* sp. This study ended with the isolation of makaluvamine O (**196**). This compound demonstrated activity with IC_50_ values of 71, 79, 94, and 8.6 µM against p53^+/+^, p53^−/−^, p21^+/+^, and p21^−/−^ cell lines [63]. Makaluvamine P (**197**), was reported from *Zyzzya* cf. *fuliginosa*, collected from the coasts of Vanuatu Islands. Compound **197** demonstrated an inhibitory effect on the KB cell line with IC_50_ values of 1.4 µM [88]. Investigation of the Caribbean sponge *Batzella* sp., yielded eight cytotoxic prroloiminoquinone alkaloids that identified as batzelline A (**198**), batzelline B (**199**), isobatzelline A (**200**), isobatzelline C (**201**), isobatzelline D (**202**), isobatzelline E (**203**), secobatzelline A (**204**), and secobatzelline B (**205**). The results proved the cytotoxic activity of **198–205** against four diverse cell lines: Panc-1, AsPC-1, BxPC-3, and MIA-PaCa2. Compounds **200–203** displayed cytotoxicity in all pancreatic cell lines with IC_50_ of less than 10 μM. Also, their cytotoxic activities on AsPC-1, BxPC3, and MIA PaCa2 had greater activity in comparison with 5-fluorouracil, a current treatment for pancreatic cancer. Interestingly, the batzellines showed less cytotoxicity in the Vero cells, which is a preferential cytotoxic ability towards tumor cell lines [89]. Figure 14 illustrates the chemical structures of **167–205**; Table 14 summarizes the cytotoxic evaluation of **167–205**.

### 2.15. Quinoline and Quinolizinde Alkaloids

**Figure 15:**
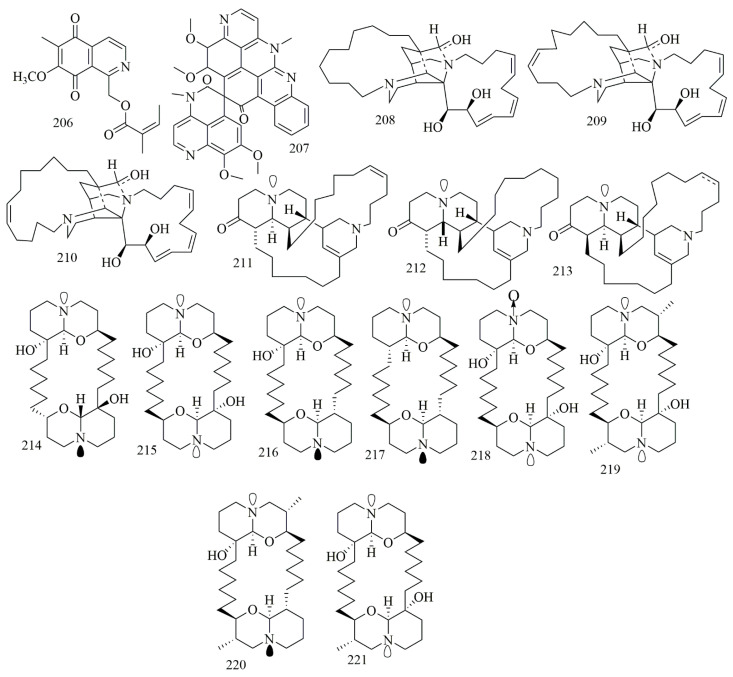
Quinoline and quinolizidine alkaloid structures in sponges.

Renierol (**206**), a cytotoxic quinoline alkaloid, was isolated from the blue hard sponge *Xestospongia* collected at Sand Island, Suva Harbor, Fiji. Compound **206** had displayed potent cytotoxic activity against L1210 cell line with IC_50_ of 9.5 µM [90]. Lihouidine (**207**), is a spiro nonacyclic polyaromatic cytotoxic alkaloid isolated from *Suberea* sponge, obtained from Lihou reef in the Coral Sea, showed moderate cytotoxicity against P388D with IC_50_ 3 µg/mL (5 µM) [91]. A specimen of *Reniera sarai* collected from the Naples gulf was the source of six quinolizidine alkaloids identified as saraine A (**208**), saraine B (**209**), saraine C (**210**), saraine 1(**211**), saraine 2 (**212**), and saraine 3 (**213**). Compounds **208–214** displayed preliminary cytotoxicity in the brine shrimp cytotoxic bioassay, revealing that compounds **211–213** were more powerfully cytotoxic (2.5 < LD50 > 6.4 μg/mL) than **208–210** (4.5 < LD50 > 46.7 μg/mL) [92]. Compounds **208–213** could also be classified as piperidine alkaloids. Eight cytotoxic macrocyclic bis-quinolizidine alkaloids **214–221** were identified from *Xestospongia muta*, collected off the coral reef of Vinh Moc, Quang Tri, Vietnam. The other isolated compounds were identified as meso-araguspongine C (**214**) araguspongines A, C, E, L, N−P (**215–221**). The result of cytotoxic assay proved **214–221** their cytotoxic activities against human cancer cell lines HepG-2, HL-60, LU-1, MCF-7, and SK-Mel-2. Stereochemistry of **216** and its meso-isomer **214** were established by NMRspectroscopy and conformational analyses. Results showed outstanding cytotoxic activities with IC_50_ values of the range of 0.43 ± 0.03 to 1.02 ± 0.11 μM, compared to positive control ellipticine (IC_50_ value of 1.18 ± 0.12 to 1.91 ± 0.28 μM) [93]. Figure 15 illustrates the chemical structures of **206–221**; Table 15 summarizes the cytotoxic evaluation of **206–221**.

### 2.16. Tetrahydroisoqouinoline Alkaloids

**Figure 16:**
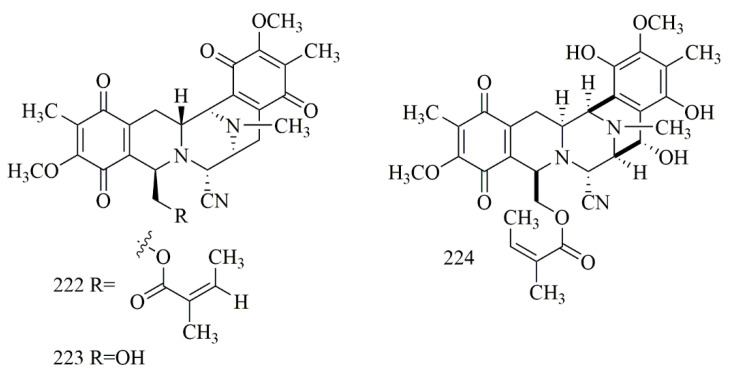
Tetrahydroisoqouinoline alkaloid structures isolated from sponges.

Based on the study by Sirimangkalakitti et al., bistetrahydroisoquinoline alkaloid, including renieramycin M (**222**), are mainly identified from sponges of the genera Reniera, Haliclona, Xestospongia, Neopetrosia, and Cribrochalina. Other renieramycin-type alkaloids, such as jorunnamycin A (**223**), were purified from the sponge-eating nudibranch *Jorunnaf unebris*, **222–223** were also reported from the Thai blue sponge *Xestospongia* sp., collected off Sichang island, the Gulf of Thailand. Compounds **222–223** revealed outstanding cytotoxic activity versus H292 cell line with IC_50_ (23 ± 4 and 220 ± 20 nM, respectively) and H460 cell line with IC_50_ 8.3 ± 0.6 and 160 ± 10 nM, respectively [94]. Renieramycin J (**224**), which has been reported from *Neopetrosia* sp., collected off Kuchinoerabu-jima island also showed outstanding cytotoxicity on 3Y1, HeLa, and P388 cells with IC_50_ values 5.3, 12.3, and 0.53 nM, respectively [95]. Figure 16 illustrates the chemical structures of **222–224**; Table 16 summarizes the cytotoxic evaluation of **222–224**.

### 2.17. Steroidal Alkaloid

**Figure 17:**
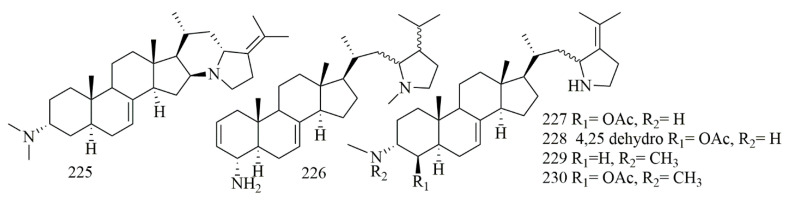
Steroid alkaloid structures isolated from marine sponges.

Four steroidal alkaloids have been reported from the Philippine sponge *Corticium niger*, collected off Boracay island. The compounds were elucidated by interpretation of spectroscopic data as plakinamine I-K (**225–227**) and dihydroplakinamine K (**228**). Compounds **225–228** showed potent cytotoxicity against HCT-116 cell lines, while **227** and **228** exhibited 1.4 µM as an IC_50_ value, **225** and **226** showed IC_50_ 10.6 and 6.1 µM, respectively [96]. Sunassee et al. investigated the same sponge, which yielded more steroidal alkaloids plakinamine N (**229**) and plakinamine O (**230**), along with **225** and **226**. Compounds **229**, **230**, and **226** were tested for antiproliferative activity in the NCI-60 panel. The test showed enhanced inhibitory effects versus all of the colon cell lines with mean GI_50_ values of 11.5, 2.4, and 1.4 μM, respectively [97]. Figure 17 illustrates the chemical structures of **225–230**; Table 17 summarizes the cytotoxic evaluation of **225–230**.

### 2.18. Manzamine Alkaloids

**Figure 18:**
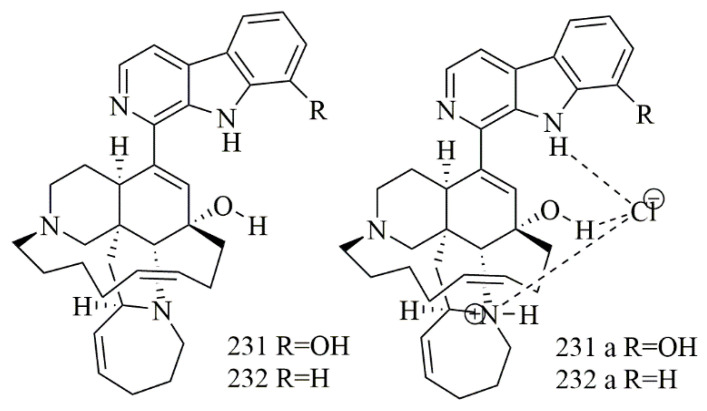
Manzamine alkaloid structures isolated from sponges.

Samoylenko et al. have reported two tertiary bases, (+)-8-hydroxymanzamine A (**231**) and (+)-manzamine A (**232**). In addition to their hydrochloride salts **231a** and **232a**, from *Acanthostrongylophora ingens* known for the biosynthesis of broad range of manzamine alkaloids as natural hydrochloride salts. Cytotoxic activity of **231**, **232** and their salts versus a panel of cell lines were tested. The hydrochloride salt **231a** was more toxic to HepG2, in comparison with **232** with IC_50_ 1.55 vs. 4.4 μg/mL, as well as being 3-fold more toxic than **232** towards kidney epithelial (non-cancer) cells (IC_50_ 0.69 vs. 2.15 μg/mL) [98]. Figure 18 illustrates the chemical structures of **231–232**; Table 18 summarizes the cytotoxic evaluation of **231–232**.

### 2.19. Diterpene Alkaloid

**Figure 19:**
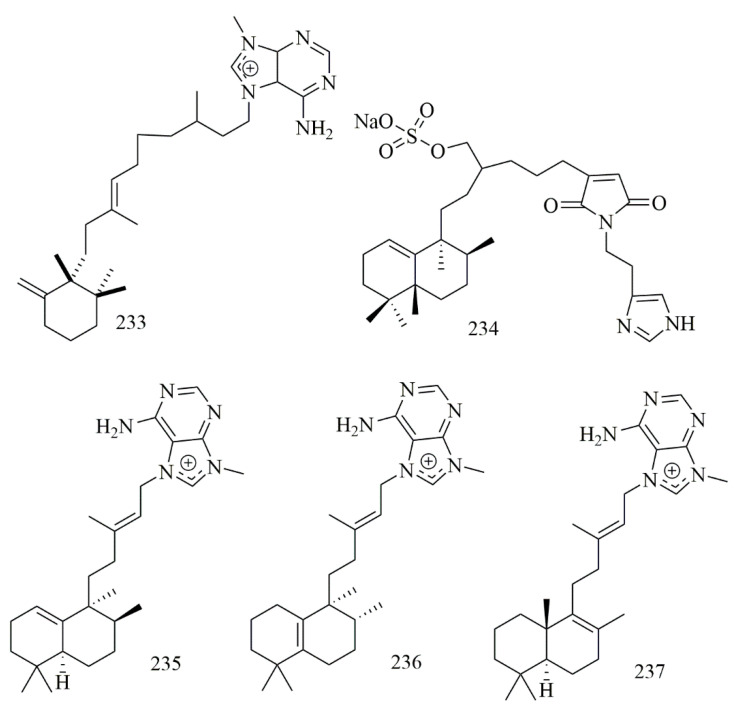
Diterpen alkaloid structures isolated from sponges.

Paige Stout et al. isolated nine diterpene alkaloids from the Caribbean sponge *Agelas citrina*, among which only agelasine E (**233**) showed potent cytotoxicity against CLL cell line with IC_50_ = 10 μM [99]. Guangmin Yao et al. isolated cytotoxic sulfated sesterterpene alkaloid 19-oxofasciospongine A (**234**) from *Fasciospongia* sp., and **234** demonstrated cytotoxic effect versus LNCaP, LU-1, and MCF-7 cell lines with IC_50_ 21.8, 5.0 and 13.4 µM, respectively [100]. Chu and coworkers reported three cytotoxic diterpene alkaloids iso-agelasine C (**235**) agelasine J (**236**), and nemoechine G (**237**) from *Agelas nakamurai* collected off the South China Sea. The results confirmed the cytotoxicity of **235** versus HCT-116, K562, and HL-60, cell lines with IC_50_ values of 19.8, 16.0, and 12.4µM, respectively [101]. Figure 19 illustrates the chemical structures of **233–237**; Table 19 summarizes the cytotoxic evaluation of **233–237**.

### 2.20. Sesquiterpene Quinones/Hydroquinones Alkaloid

**Figure 20:**
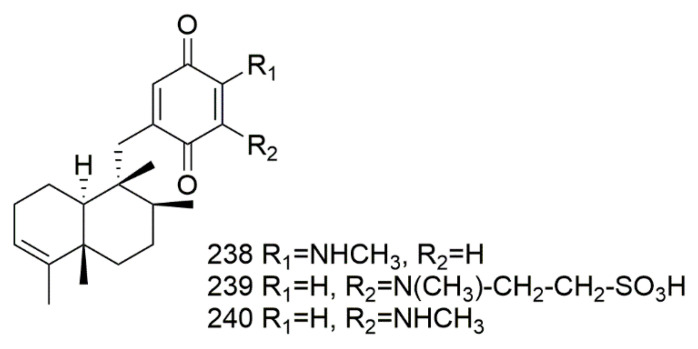
Sesquiterpene quinones/hydroquinones alkaloid structures isolated from sponges.

The sponge *Dysidea avara* is a great source of sesquiterpene quinones/hydroquinone alkaloid, which the isolated compounds showed antileishmanial, antiplasmodial, antischistosomal, and cytotoxic activity [102]. The sesquiterpene(−)-4’-methylaminoavarone (**238**). (−)-N-methylmelemeleone-A (**239**), and (−)-3’-methylaminoavarone (**240**) isolated from *Dysidea avara* collected from the Mediterranean Sea in Fethiye, Turkey. The results revealed that compound **240** proved to be highly toxic, while **239** displayed very weak cytotoxic activity L5178Y cell line. The results from HCT116 cells revealed weak toxicity for **238–240** were IC50-values of 9,> 50, 45 µM, respectively. Results from H4IIE cells exhibited similar results—**239** demonstrated weak cytotoxic activity with IC50- value more than 50 µM, while **238** and **240** showed IC50-values of 40 and25 µM, respectively [103]. Figure 20 illustrates the chemical structures of **238–240**; Table 20 summarizes the cytotoxic evaluation of **238–240**.

## 3. Conclusions

Isolation and identification of new cytotoxic alkaloids extracted from marine animals, particularly sponges, is a multidisciplinary and intricate endeavor. This is because of the lack of knowledge on traditional experiential and ethnopharmacology investigations that have endorsed this zoochemical scrutiny. Hence, not only is it fundamental to assemble a database to address the shortcomings in the scholarly publication, but it is also bound to be on natural products identification. The representative survey depicted the study on marine cytotoxic alkaloid originated from sponge accomplished between 1987 and 2020 in different resources and databases had been investigated, including Scifinder (database for the chemical literature) CAS (Chemical Abstract Service) search, web of science, Marin Lit (marine natural products research) database. This investigation ended in 80 closely-correlated articles, certifying, as an example, the considerable chemodiversity of sponges as original of secondary cytotoxic metabolite. During these 33 years database search, natural products originated, including novel, new, and known compounds, were reported as cytotoxic chemical structures. Several of these structures have demonstrated authenticated in vitro cytotoxic bioactivities versus a panel of cancer/tumor cell lines. In addition, several of them are attracting the interest for future in vivo assay. The main chemical classes of these alkaloid structures were confined, particularly to the Pyrroloiminoquinone alkaloids (16.4%), Guanidine alkaloids (13.9%), Indole alkaloids (12.6%), Pyridine alkaloids (10.6%), and Bromotyrosine alkaloids (8.9%)—demonstrating the five major chemical classes of alkaloid structures observed in sponges during this time, which along with other chemical classes as Quinolizidine and Brominated alkaloids, and others (47.6%), exhibited a various range of cytotoxicity activity. Verongida, Poecilosclerida, Agelasida, Haplosclerida, Halichondrida, Dictyonellidae, and Homosclerophorida were the sponge’s main orders in this review, and were also discovered to be the most prosperous genera, due to their potential to produce promising bioactive alkaloid compounds. The examination of the contribution from an individual species revealed that regardless of the order, each species contributed, on average, 2–5 new compounds. In addition, alkaloids with cytotoxic activities have been isolated from 49 genus of sponges. Genus *Agelas* was discovered to be the most lucrative producer of potent alkaloid compounds, then *Biemna*, *Suberea*, and *Pachychalina* were established. The geniuses *Mycale*, *kirkpatrickia*, *Scleritoderma*, *Zyzzya*, and *Dercitus* only had one cytotoxic alkaloid. Among the reported alkaloid in this review, Dercitine (acridin alkaloid), Crambescidin 814 (guanidine alkaloid), Crambescidin 816 (guanidine alkaloid), Unguiculin A-C (guanidine alkaloid), Dihydrodiscorhabdin A and L (Pyrroloiminoquinone alkaloid), Discorhabdin A (Pyrroloiminoquinone alkaloid), Renierol (quinolone alkaloid), Renieramycin(quinolone alkaloid), Renieramycin J (quinolone alkaloid) and Jurunnamycin A (quinolone alkaloid) showed the best cytotoxic activity in micro molar to nano molar ranges.

Evaluation of cytotoxicity is requisite to approach the cytotoxic level of alkaloid structures. In this survey, the evaluation of cytotoxicity of the alkaloid compounds, extracted from marine sponges, was determined through the most usual panels of cytotoxic cell lines—comprised of either in human [breast cancer (MCF-7 and MDA-MB-231), colorectal carcinoma HCT-116, HCT15, HCT8, Sw620, and HCC-2998), alveolar adenocarcinomic (A549), lung Adenocarcinoma (PC9), lung fibroblast (MALME3M), fibroblast (FS4-LTM and MRC-5), leukemia (THP-1, HL-60, K562, MOLT-4, and CCRF-CEM), cervical cells (Hela), ovarian cell line (A2780, OVCAR-3 and SK-OV-3), melanoma (SK-MEL-2), renal (A498 and UO-31), melanoma (SK-MEL-2 and MM.1S), Glioblastoma (U138 and SF-295), liver (HepG2 and Huh7), PSN1 (pancreatic), and CNS solid tumors (XF498)], or murine [lymphocytic leukemia (L1210and P388D1); melanoma (B16 and B16F10)] cancerous cell-lines.

Nevertheless, in infrequent studies were wide-range and have consisted of the NCI-60 panel of cell lines for screening anticancer characteristics of the drug. These kinds of screening system are great implements for clarifying the relationship between cytotoxicity and anticancer drugs, for the exploration of molecular-targeted antineoplastic drugs, and improve the compounds, selection as candidates for antineoplastic drug. Even though it was not the object of this survey to consider preclinical and clinical perspective of the cytotoxic isolated alkaloids, it is obvious that the present gap with regard to the natural approachability of these cytotoxic alkaloids to carry out studies, since they need higher amounts of compounds. As far as the amounts of substances are concerned, preparing synthetic compounds influences the preclinical phase for drug candidates. Synthesis of natural products and their chemical derivatives display the potential to assert or modify isolated alkaloids, as well as to prepare effective analogs. Sponge aquatic environment and microorganisms engineering are attracted interesting to support these kinds of compounds. Sufficient production of alkaloids derived from sponges is a prosperous approach that requires more attention in future to consider the constraints regarding the supply of drugs, attained from marine organism. In conclusion, this review is able to point out the importance of cytotoxic alkaloids to explore new, effective anticancer drugs.

## Figures and Tables

**Table 1:** Cytotoxic activity of acridine alkaloids.

	Compound	Cell Line (IC50 values µM)	Source	Place of Collection	Ref.
**1**	dercitin	P388 = 0.081 A-549 = 0.075 HT-29 = 0.063 HL-60 = 0.150 HL-60/AR = 0.240	Dercitus sp.	Bahamas	[23]
**2**	neoamphimedine	L1210 = 7.6 C38 = 7.6 H116 = 7.6 H125 = 7.6 CEM = 7.6 CFU-GM = 7.6	Xestospongia sp.	Indonesia and Papua	[24]
**3**	5-methoxyneoamphimedine	L1210 = 72.8 C38 = 72.8 H116 = 72.8 H125 = 72.8 CEM = 72.8 CFU-GM = 72.8			
**4**	amphimedine	L1210 = 11.9 C38 = 11.9 CFU-GM = 11.9			
**5**	neoamphimedine Z	-			
**6**	alpkinidine	L1210 = 362 C38 = 362 CFU-GM = 362			

**Table 2:** Cytotoxic activity of β-carboline alkaloids.

	Compound	Cell Line (IC50 µM)	Source	Place of Collection	Ref.
**7**	1,2,3,4-tetrahydronorharman-1-one	MCF7 = 44.4 HCT116 = 40.0 A549 = 54.3	*Acanthostrongylophora ingens*	Sulawesi Island in Indonesia	[25]
**8**	acanthomine A2	MCF7 = 10.6 HCT116 = 2.2 A549 = 7.3			
**9**	annomontine1	MCF7 = 4.6 HCT116 = 1.5 A549 = 4.1			
**10**	ingenine E	MCF7 = 13.1 HCT116 = 2.5 A549 = 8.0			

**Table 3:** Cytotoxic effects of bromotyrosine alkaloids.

	Compound	Cell Line (IC50 µM)	Source	Place of Collection	Ref.
**11**	14-debromo-11-deoxyfistularin-3	KB-31 = 68.8	*Aplysina lacunosa*	Bahamas	[26]
**12**	aplysinin A	KB-31 = 25.8 MCF-7 = 77.5 FS4-LTM = 32.2			
**13**	aplysinin B	-			
**14**	aplyzanzine B	A549 = 6.1 HT-29 = 1.6 MDA-MB-231 = 7.8	*Jaspis* sp. and *Bubaris* sp.	Indonesia	[27]
**15**	anomoian B	A549 = 5.1 HT-29 = 3.2 MDA-MB-231 = 5.3	*Hexadella* sp.	Indonesia	
**16**	ma’edamines C	L1210 = 12.3	*Suberea* sp.	Okinawa	[28]
**17**	ma’edaminesD	L1210 = 5.0			
**18**	psammaplysene C	THP-1 = 7	*Psammoclemma* sp.	Australia	[29]
**19**	psammaplysene D	THP-1 = 7			
**20**	purealidin Q	A2780 = 3.4 K562 = 2	*Druinella* sp.	Fiji Islands	[30]
**21**	purealidin S	A2780 = 10.2 K562 = 8.02			
**22**	aplysamine 2	A2780 = 4.3 K562 = 2.1			
**23**	purpureamine I	A2780 = 2.6 K562 = 1.9			
**24**	purpureamine J	A2780 = 10.2 K562 = 9.0			
**25**	aerophobin 2	K562 = 13.7			
**26**	aerophobin 1	A2780 = 45.5 K562 = 50.9			
**27**	purealidin J	A2780 > 20.4 K562 > 20.4			
**28**	araplysillin 1	A2780 = 26.0 K562 = 39.2			
**29**	araplysillin 2	A2780 = 15.7 K562 = 45.5			
**30**	suberedamines A	L1210 = 12.6 KB = 14.2	*Suberea* sp.	Okinawa	[32]
**31**	suberedamines B	L1210 = 13.2			

**Table 4:** Cytotoxic activity of dibrominated and brominated alkaloids from sponges.

	Compound	Cell Line (IC_50_ µM)	Source	Place of Collection	Ref.
**32**	agelastatin A	KB = 3	*Agelas* *dendromorpha*	New Caledonia	[32]
**33**	aerothionin	MDA-MB-231 > 50 Hella = 29	*Suberea* sp.	Saudi Red Sea	[33]
**34**	echinosulfonic Acid D	KB = 3.7	*Psammoclemma* sp.	New Caledonia	[34]
**35**	echinosulfonic Acid B	KB = 3.5			

**Table 5:** Cytotoxic activity of Dimeric aaptamine alkaloids.

	Compound	Cell Line (IC_50_ µM)	Source	Place of Collection	Ref.
**36**	aaptamine	Hela = 65.7	*Aaptos suberitoides*	Indonesia	[35]
**37**	isoaaptamine	Hela = 13.5			
**38**	demethylaaptamine	Hela = 6.5			
**39**	suberitine A	P388 < 50% at 10 HeLa < 50% at 10 K562 < 50% at 10	*Aaptos suberitoides*	South China Sea	[36]
**40**	suberitine B	P388 = 1.8 HeLa < 50% at 10 K562 < 50% at 10			
**41**	suberitine C	P388 < 50% at 10 HeLa < 50% at 10 K562 < 50% at 10			
**42**	suberitine D	P388 = 3.5 HeLa < 50% at 10 K562 < 50% at 10			

**Table 6:** Cytotoxic activity of Guanidine alkaloids from marine sponges.

	Compound	Cell Line (IC50 µM)	Source	Place of Collection	Ref.
**43**	monanchocidin A	THP-1 = 5.1 HeLa = 11.8 JB6 Cl41 = 12.3 HL60 = 0.540	*Monanchora* *pulchra*	Indonesia	[38,39]
**44**	monanchocidin B	HL60 = 0.200	*Monanchora* *pulchra*	Indonesia	[39]
**45**	monanchocidin C	HL60 = 0.110			
**46**	monanchocidin D	HL60 = 0.830			
**47**	monanchocidin E	HL60 = 0.650			
**48**	monanchoradin A	HCT-116 = 9.9/9.9 MDA-435 = 11/9.3 HL-60 = 3.8/7.1	*Monanchora* *pulchra*	Indonesia	[40]
**49**	dehydrocrambescin A2 418	KB = 0.1/0.1 HCT-116 = 3.4/3.5 HL-60 = 3.6/5.4 MRC-5 = 3.4/3.9			
**50**	crambescidin 786	KB = 0.3/0.3 HCT-116 = 3.1/3.4 HL-60 = 5.0/5.4 MRC-5 = 3.2/3.4			
**51**	(−)-crambescidin 814	KB = 0.005/0.02 HCT-116 = 0.02/0.05 MDA-435 = 0.04/0.07 HL-60 = 0.01/0.03 B16-F10 = 0.20			
**52**	monalidin A	HCT-116 = 0.84/0.74 MDA-435 = 0.32/0.86 HL-60 = 1.3/1.3 MRC-5 = 0.55/0.60			
**53**	crambescidin 406	HCT-116 = 3.4/4.2 HL-60 = 8.0/9.1 MRC-5 = 4.1/3.6			
**54**	crambescidin 800	HCT-116 = 0.007 MDA-435 = 0.009/0.015 HL-60 = 0.004/0.006 B16-F10 = 0.2 A431 = 3.1			
**55**	crambescidin 826	B16-F10 = 0.8			
**56**	20-norcrambescidic acid	KB = 0.5/0.6			
**57**	crambescidin 816	HepG2 = 0.150	*Crambe crambe*	Indonesia	[41]
**58**	crambescidins 345	A431 = 0.012	*Clathria bulbotoxa*	Samalona Island, South Sulawesi, Indonesia	[43]
**59**	crambescidins 361	A431 = 0.048			
**60**	crambescidins 373	A431 = 0.007			
**61**	crambescidins 359	A431 = 0.0025			
**62**	crambescidins 657	A431 = 0.094			
**63**	normonanchocidins A	THP-1 = 2.1 HeLa = 3.7	*Monanchora* *pulchra*	Urup Island	[44]
**64**	normonanchocidins B	THP-1 = 3.8 HeLa = 6.8			
**65**	normonanchocidins D	THP-1 = 3.8 HeLa = 6.8			
**66**	monanchomycalin C	MDA-MB-231 = 8.2	*Monanchora* *pulchra*	Kunashir Island	[45]
**67**	ptilomycalin A	MDA-MB-231 = 4.3	*Monanchora* *pulchra*, *Ptilocaulis* *spiculifer*, *Hemimycale* sp., *M. arbuscula*, *M. unguifera*	Kunashir Island	
**68**	monanchoxymycalin C	Hella = 3.5	*Monanchora* *pulchra*	Chirpoi Island	[46]
**69**	netamines M	KB = 1	*Biemna laboutei*	Salary Bay, Madagascar	[48]
**70**	netamines O	KB = 10			
**71**	netamines Q	KB = 10			
**72**	zanissine	P-388 = 88.8 KB = 37.0 NSCLC-N6 = 74.0	*Anchinoe pauperta*	Zarzis, Tunisia	[49]
**73**	unguiculin A	KB = 0.2	*Monanchora* sp.	Hiva Oa Island (French Polynesia)	[50]
**74**	unguiculin B	KB = 0.08 HCT-116 = 3.6 HL-60 = 10 MRC-5 = 11.4			
**75**	unguiculin C	KB = 0.03			

**Table 7:** Cytotoxic effects of Imidazole alkaloid.

	Compound	Cell Line (IC_50_ µM)	Source	Place of Collection	Ref.
**76**	(−)-calcaridine	MCF-7 = 25.3	*Leucetta* *chagosensis*	South China Sea	[51]
**77**	(2E, 9E)-pyronaamidine-9-(N-methylimine)	MCF-7 = 24.2			
**78**	naamidine J	K562 = 11.3	*Pericharax* *heteroraphis*	South China Sea	[52,54,57]
**79**	naamidine H	K562 = 9.4 HeLa = 21.4 A549 = 22.4 Hella = 11.3			
**80**	naamine J	MCF-7 = 20.1 A549 = 23.7 HeLa = 28.2 PC9 = 45.3	*Leucandra* sp.	Woody (Yongxing) Islands in the South China Sea	[53]
**81**	naamidine I	Hella = 29.6	*Leucetta* *chagosensis*	North Sulawesi, Indonesia	[54]
**82**	isonaamine C	HM02 = 15.0 HepG2 = 6.2 Huh7 = 5.9	*Leucetta* *chagosensis*	Bougainville Reef, Australia	[55]
**83**	isonaamidine E	HM02 = 15.1 HepG2 = 15.1 Huh7 = 2.8			
**84**	leucosolenamine B	C-38 = 19.6	*Leucosolenia* sp.	Milne Bay in Papua New Guinea	[56]
**85**	chagosendine A	K562 > 10 HepG2 > 10 Hella > 10	*Leucetta* *chagosensis*	South China Sea	[57]
**86**	chagosendine B	K562 = 0.62 HepG2 = 1.19 Hella = 0.58			
**87**	chagosendine C	K562 = 0.62 HepG2 = 0.31 Hella = 4.43			
**88**	pyronaamidine	K562 = 6.57 Hella = 5.62 K562 = 6.87 Hella = 5.62			

**Table 8:** Cytotoxic activity of Indol, Bisindol, and Trisindol alkaloids.

	Compound	Cell Line (IC_50_ µM)	Source	Place of Collection	Ref.
**89**	demethoxyfascaplysin	MCF-7 = 20.4	*Thorectandra* sp.	Palau	[58]
**90**	1-deoxysecofascaplysin A	MCF-7 = 4.9 OVCAR-3 = 7.2 A549 = 43.2			
**91**	fascaplysin	0.11 ˂ MCF-7 ˂ 1.4 0.11 ˂ OVCAR-3 ˂ 1.4 0.11 ˂ MALME- 3M ˂ 1.4 0.11 ˂ A549 ˂ 1.4			
**92**	dragmacidin G	Hella = 4.2	*Lipastrotethya* sp.	Japan	[59]
**93**	dragmacidin H	Hella = 4.6			
**94**	topsentin B1	Hella = 4.4			
**95**	topsentin B2	Hella = 1.7			
**96**	hyrtinadine A	L1210 = 2.9 KB = 8.7	*Hyrtios* sp.	Japan	[60]
**97**	hyrtioerectine A	Hela = 25.8	*Hyrtios erectus*	Red Sea	[61]
**98**	hyrtioerectine B	Hela = 20.3			
**99**	hyrtioerectine C	Hela = 20.4			
**100**	5-bromotrisindoline	HT-29 = 8 OVCAR-3 = 7 MM.1S = 9	*Callyspongia* *siphonella*	Red sea	[62]
**101**	6-bromotrisindoline	HT-29 = 12.5 OVCAR-3 = 9 MM.1S = 11			
**102**	5-bromo-l-tryptophan	p53^+/+^ > 177 p53^−/−^ > 177	*Smenospongia* sp.	Batanes, Philippines.	[63]
**103**	5-bromoabrine	p53^+/+^ > 168 p53^−/−^ > 168			
**104**	5,6-dibromoabrine	p53^+/+^ > 133 p53^−/−^ > 133			
**105**	5-bromoindole-3-acetic acid	p53^+/+^ > 197 p53^−/−^ > 197			
**106**	damirine A	MALME-3M = 1.9 Sw620 = 3.3 HCC-2998 = 2.3 MOLT-4 = 1.9 k562 = 2.2	*Damiria* sp.	Thailand	[64]
**107**	6″debromohamacanthin A	eMs = 28.5	*Spongosorites* sp.	Korea	[65]
**108**	(R)-6′′-debromohamacanthin A	A549 = 13.7 SK-OV-3 = 10.2 SK-MEL-2 = 11.5 XF498 = 10.0 HCT15 = 8.7	*Spongosorites* sp.	Korea	[66]
**109**	(R)-6′-debromohamacanthin A	A549 > 73.7 SK-OV-3 > 73.7 SK-MEL-2 > 73.7 XF498 > 73.7 HCT15 = 65.7			
**110**	(S)-6′′-debromohamacanthin B	A549 > 73.7 SK-OV-3 > 73.7 SK-MEL-2 > 73.7 XF498 > 73.7 HCT15 > 73.7			
**111**	dibromodeoxytopsentin	A549 > 61.8 SK-OV-3 > 61.8 SK-MEL-2 > 61.8 XF498 > 61.8 HCT15 > 61.8			
**112**	trans-3,4-dihydrohamacanthin A	A549 = 16.9 SK-OV-3 = 16.4 SK-MEL-2 = 18.7 XF498 = 14.0 HCT15 = 10.9			
**113**	cis-3,4-dihydrohamacanthin B	A549 = 7.0 SK-OV-3 = 7.4 SK-MEL-2 = 7.9 XF498 = 6.6 HCT15 = 5.8			
**114**	topsentin	A549 > 87.4 SK-OV-3 > 87.4 SK-MEL-2 > 87.4 XF498 > 87.4 HCT15 = 38.8 P388 = 5.8	*Spongosorites* sp.	Jeju Island, Korea	[66]
**115**	bromotopsentin	A549 => 30.0 SK-OV-3 = 28.14 SK-MEL-2 = 7.02 XF498 = 14.99 HCT15 > 30.0 P388 = 7.0			
**116**	deoxytopsentin	A549 > 70.9 SK-OV-3 > 70.9 SK-MEL-2 > 70.9 XF498 > 70.9 HCT15 = 61.8			
**117**	bromodeoxytopsentin	A549 > 30.0 SK-OV-3 > 30.0 SK-MEL-2 > 30.0 XF498 > 30.0 HCT15 = 11.48 K562 = 0.6			
**118**	isobromodeoxytopsentin	A549 = 30.2 SK-OV-3 = 21.3 SK-MEL-2 = 11.1 XF498 = 13.5 HCT15 = 15.6 K562 = 5.1			

**Table 9:** Cytotoxic activity of peptide alkaloid.

	Compound	Cell Line (IC50 µM)	Source	Place of Collection	Ref.
**119**	scleritodermin A	HCT116 = 1.9 A2780 = 0.940 SKBR3 = 0.670	*Scleritoderma nodosum*	Philippines	[67]

**Table 10:** Cytotoxic activity of piperidine alkaloid.

	**Compound**	**Cell Line** **(IC_50_ µM)**	**Source**	**Place of** **Collection**	**Ref.**
**120**	arenosclerins A	HL-60 = 8.9 B16 = 3.6 L929 = 4.8 U138 = 7.9	*Arenosclera* *brasiliensis*	Brazil	[68]
**121**	arenosclerins B	HL-60 = 8.4 B16 = 3.6 L929 = 4.6 U138 = 7.5			
**122**	arenosclerins C	HL-60 = 7.5 B16 = 3.5 L929 = 4.5 U138 = 7.4			
**123**	haliclonacyclamine E	HL-60 = 9.0 B16 = 3.9 L929 = 8.3 U138 = 13.0			
**124**	madangamine F	SF295 = 41.4 MDA-MB435 = 33.8 HCT8 > 52.3 HL60 = 34.9	*Pachychalina* *alcaloidifera*		[69]
**125**	haliclonacyclamine F	SF295 = 9.6 MDA-MB435 = 2.1 HCT8 = 18.4 HL60 = 4.7			
**126**	arenosclerins D	SF295 = 12.2 MDA-MB435 = 2.4 HCT8 = 12.8 HL60 = 4.3			
**127**	arenosclerins E	SF295 = 18.0 MDA-MB435 = 6.4 HCT8 > 51.8 HL60 = 14.3			
**128**	neopetrosiamine A	MALME-3M = 1.5 CCRF-CEM = 2.0 MCF-7 = 3.5	*Neopetrosia* *proxima*	Puerto Rico	[70]
**129**	1,5-diazacyclohenicosane	A549 = 5.41 HT29 = 5.07 MDA-MB-231 = 5.74	*Mycale* sp.	Kenya	[71]
**130**	ingenamine G	HCT-8 = 17.9 B16 = 20.5 MCF-7 = 23.6	*Pachychalina* sp.	Rio de Janeiro	[72]
**131**	papuamine	UO-31 = 3.0 A498 = 2.9 SF-295 = 0.8 and MCF-7 = 20 μM concentration at 3, 6, 12 and 24 h the cell survival is significantly reduced 40.0 ± 15.3%, 11.3 ± 10.4%, 5.2 ± 2.7% and 1.3 ± 1.1%	*Neopetrosia cf* *exigua* and *Haliclona* sp.	Indonesia	[73,74]
**132**	haliclonadiamine	UO-31 = 8.0 A498 = 5.9 SF-295 = 6.3	*Haliclona* sp.	Indonesia	[73]

**Table 11:** Cytotoxic activity of pyrimidine alkaloids from sponges.

	Compound	Cell Line (IC_50_ µM)	Source	Place of Collection	Ref.
**133**	lanesoic acid	PSN1 = 28.2	*Theonella swinhoei*	Indonesia	[75]
**134**	variolin B	DU-145 = 0.89 LN-caP = 0.05 SKOV-3 = 1.21 IGROV = 1.14 IGROV-ET = 1.28 SK-BR-3 = 0.85 MEL-28 = 1.20 H-MEC-1 = 0.27 A-549 = 0.98 K-562 = 1.55 PANC-1 = 1.68 HT-29 = 2.85 LOVO = 0.80 LOVO-DOX = 1.02	*Kirkpatrickia* *variolosa*		[76]

**Table 12:** Cytotoxic activity of pyridine alkaloids.

	Compound	Cell Line (IC_50_ µM)	Source	Place of Collection	Ref.
**135**	3-dodecyl pyridine along with terminal cyano entity	A549 = 41.8 MCF-7 = 48.4 Hela = 33.2	*Haliclona* sp.	Indonesia	[77]
**136**	amphimedoside A	P388 = 21.7	*Amphimedon* sp	Japan	[78]
**137**	amphimedoside B	P388 = 23.0			
**138**	amphimedoside C	P388 = 10.4			
**139**	amphimedoside D	P388 = 0.9			
**140**	amphimedoside E	P388 = 4.5			
**141**	hachijodine A	P388 = 7.5	*Xestospongia* sp.	Japan	[79]
**142**	hachijodine B	P388 = 7.1			
**143**	hachijodine C	P388 = 7.1			
**144**	hachijodine D	P388 = 7.1			
**145**	hachijodine E	P388 = 7.4	*Amphimedon* sp.		
**146**	hachijodine F	P388 = 3.1			
**147**	hachijodine G	P388 = 2.9			
**148**	N-methylniphatyne A	PANC-1 = 16	Xestospongia sp.	Indonesia	[80]
**149**	niphatyne A	P388 = 2			
**150**	pyrinodemin A	L1210, KB > 17.4	*Amphimedon* sp.	Okinawa	[81]
**151**	pyrinodemin B	L1210 = 0.12 KB = 0.89			
**152**	pyrinodemin C	L1210 = 0.1 KB = 0.89			
**153**	pyrinodemin D	L1210 = 0.14 KB = 0.91			
**154**	pyrinadine B	L1210 = 23.0 KB > 34.9	*Cribrochalina* sp.	Okinawa	[82]
**155**	pyrinadine C	L1210 = 18.1 KB > 36.3			
**156**	pyrinadines D	L1210 = 17.3 KB > 34.7			
**157**	pyrinadines E	L1210 = 16.0 KB > 35.5			
**158**	pyrinadines F	L1210 = 11.9 KB > 34.0			
**159**	pyrinadines G	L1210 = 11.9 KB > 34.0			

**Table 13:** Cytotoxic effects of pyrrole and bromopyrrole alkaloids.

	**Compound**	**Cell Line (IC_50_ µM)**	**Source**	**Place of** **Collection**	**Ref.**
**160**	(−)-clathramide C	L5178Y = 25.3%	*Stylissa carteri*	Red Sea, Hurghada, Egypt	[83]
**161**	(+)-dibromophakelline	L5178Y = 57%			
**162**	(*Z*)-spongiacidin D	L5178Y = 36.7%			
**163**	(*Z*)-hymenialdisine	L5178Y = 37% HCT116 = 25			
**164**	(*Z*)- 3-bromohymenialdisine	L5178Y = 60.5% HCT116 = 25			
**165**	3,4-dibromo-1H-pyrrole-2-carbamide	L5178Y = 38.4%			
**166**	oroidin	* HT29 = 31 SW480 = 8 MCF-7 = 27 A2780 = 51 H460 = 7 A431 = 6 Du145 = 23 BE2-C = 6 MIA = 21 SMA = 15 U87 = 3	*Agelas oroides*		[84]

* (%) growth inhibition in response to 25 µM.

**Table 14:** Cytotoxic activity of pyrroloiminoquinone alkaloids in sponges.

	Compound	Cell Line (IC_50_ µM)	Source	Place of Collection	Ref.
**167**	14-bromodiscorhabdin	HCT-116 = 0.077	*Tsitsikamm* *apedunculaa*	South Africa	[85]
**168**	14-bromo-3-dihydrodiscorhabdin C	HCT-116 = 0.645			
**169**	3-dihydrodiscorhabdin C	HCT-116 = 0.323			
**170**	3-dihydro-7,8-dehydrodiscorhabdin C	HCT-116 = 0.197			
**171**	14-bromo-3-dihydro-7,8-dehydrodiscorhabdin C	HCT-116 = 0.222			
**172**	discorhabdin V	HCT-116 = 1.266			
**173**	14-bromo-1-hydroxydiscorhabdin V	HCT-116 = 12.496			
**174**	tsitsikammamine A	HCT-116 = 1.414	*Tsitsikamma* *favus*	South Africa	
**175**	tsitsikammamine B	HCT-116 = 2.382			
**176**	tsitsikammamine A N-18 oxime	HCT-116 = 128.213			
**177**	tsitsikammamine B N-18 oxime	HCT-116 = 16.541			
**178**	1-methoxydiscorhabdin D	HCT-116 = 0.232	*Latrunculia bellae*	South Africa.	
**179**	1-aminodiscorhabdin D	HCT-116 = 0.119			
**180**	damirone B	HCT-116 = 3.102			
**181**	makaluvic acid A	HCT-116 = 28.399			
**182**	makaluvamine C	HCT-116 = 1.089			
**183**	discorhabdin G	HCT-116 = 0.327			
**184**	discorhabdin N	HCT-116 = 2.249			
**185**	discorhabdin A	HCT-116 = 0.007	*Strongylodesma algoaensis*	South Africa	
**186**	discorhabdin D	HCT-116 = 0.595			
**187**	discorhabdin H	-			
**188**	discorhabdins L	* HT-29 = 0.12	*Latrunculia brevis*	Argentina	[86]
**189**	discorhabdins I	* HT-29 = 0.35			
**190**	dihydrodiscorhabdin A	-	*Higginsia* sp.	Deal Island	[87]
**191**	(+)-debromodiscorhabdin A	-			
**192**	(+)-discorhabdin X	-	*Spongosorites* sp.	Port Campbell	
**193**	makaluvamine J	-			
**194**	damirone	-			
**195**	(+)-dihydrodiscorhabdin L	-			
**196**	makaluvamine O	p53^+/+^ = 71 p53^−/−^ = 79 p21^+/+^ = 94 p21^−/−^ = 8.6	*Smenospongia* sp.	Philippine	[63]
**197**	makaluvamine P	KB = 1.4	*Zyzzya* cf. *fuliginosa*	Vanuatu Islands	[88]
**198**	batzelline A	Panc-1 > 17.683 AsPC1 > 17.683 BxPC3 > 17.683 MIA-PaCa2 > 17.683	*Batzella* sp.	Madagascar	[89]
**199**	batzelline B	Panc-1 > 18.607 AsPC-1 > 18.607 BxPC-3 > 18.607 MIA-PaCa2 > 18.607			
**200**	isobatzelline A	Panc-1 = 9.37 ± 0.536 AsPC1 = 1.736 ± 0.415 BxPC3 = 2.392 ± 0.218 MIA-PaCa2 = 4.342 ± 0.22			
**201**	isobatzelline C	Panc-1 = 9.978 ± 0.384 AsPC-1 = 1.723 ± 0.168 BxPC-3 = 1.311 ± 0.185 MIA-PaCa2 = 2.343 ± 0.977			
**202**	isobatzelline D	Panc-1 = 5.723 ± 0.253 AsPC-1 = 1.477 ± 0.18 BxPC-3 = 1.48 ± 0.18 MIA-PaCa2 = 2.672 ± 0.29			
**203**	isobatzelline E	Panc-1 > 21.459 AsPC-1 > 21.459 BxPC-3 > 21.459 MIA-PaCa2 > 21.459			
**204**	secobatzelline A	Panc-1 = 10.389 ± 1.15 AsPC-1 = 3.623 ± 0.80 BxPC-3 = 4.095 ± 0.14 MIA-PaCa2 = 5.626 ± 0.739			
**205**	secobatzelline B	Panc-1 = 17.372 ± 0.281 AsPC-1 > 19.531 BxPC-3 > 19.531 MIA-PaCa2 > 19.531			

* GI_50_ values of μM.

**Table 15:** Cytotoxic activity of quinoline and quinolizidine alkaloids.

	Compound	Cell Line (IC_50_ µM)	Source	Place of Collection	Ref.
**206**	renierol	L1210 = 9.5	*Xestospongia*	Fiji	[90]
**207**	lihouidine	P388D = 4.8	*Suberea* sp.	Lihou reef	[91]
**208**	saraine A	* *Artemia salina* = 9.15	*Reniera sarai*	Naples Gulf	[92]
**209**	saraine B	* Artemia salina = 13.0			
**210**	saraine C	* Artemia salina = 8.3			
**211**	saraine 1	* Artemia salina = 7.1			
**212**	saraine 2	* Artemia salina = 14.1			
**213**	saraine 3	* Artemia salina = 5.2			
**214**	meso-araguspongine C	HepG-2 = 0.75 HL-60 = 0.88 LU-1 = 0.96 MCF-7 = 0.79 SK-Mel-2 = 1.02	*Xestospongia muta*	Vietnam	[93]
**215**	araguspongine A	HepG-2 = 0.43 HL-60 = 0.62 LU-1 = 0.76 MCF-7 = 0.44 SK-Mel-2 = 0.77			
**216**	araguspongine C	HepG-2 = 6.58 HL-60 = 7.84 LU-1 = 9.20 MCF-7 = 7.36 SK-Mel-2 = 11.23			
**217**	araguspongine E	HepG-2 = 5.06 HL-60 = 5.65 LU-1 = 5.63 MCF-7 = 5.32 SK-Mel-2 = 5.45			
**218**	araguspongine L	HepG-2 = 5.55 HL-60 = 6.58 LU-1 = 5.84 MCF-7 = 5.68 SK-Mel-2 = 6.24			
**219**	araguspongine N	HepG-2 = 6.85 HL-60 = 9.19 LU-1 = 9.88 MCF-7 = 7.82 SK-Mel-2 = 7.51			
**220**	araguspongine O	HepG-2 = 30.35 HL-60 = 22.95 LU-1 = 32.59 MCF-7 = 24.8 SK-Mel-2 = 35.92			
**221**	araguspongine P	HepG-2 = 19.52 HL-60 = 16.79 LU-1 = 22.25 MCF-7 = 24.85 SK-Mel-2 = 23.04			

* Brine shrimp.

**Table 16:** Cytotoxic activity of tetrahydroisoqouinoline alkaloids.

	Compound	Cell Line (IC_50_ nM)	Source	Place of Collection	Ref.
**222**	renieramycin M	H292 = 23 H460 = 8.3 NSCLC = 24	*Xestospongia* sp.	Thailand	[94]
**223**	jorunnamycin A	H292 = 220 H460 = 160			
**224**	renieramycin J	3Y1 = 5.3 HeLa = 12.3 P388 = 0.53	*Neopetrosia* sp.	Kuchinoerabu-jima Island	[95]

**Table 17:** Cytotoxic activity of Steroidal alkaloids.

	Compound	Cell Line (IC_50_ µM)	Source	Place of Collection	Ref.
**225**	plakinamine I	HCT-116 = 10.6	*Corticium niger*	Philippines	[96,97]
**226**	plakinamine J	HCT-116 = 6.1 NCI-60 screen = 1.4			
**227**	plakinamine K	HCT-116 = 1.4			
**228**	dihydroplakinamine K	HCT-116 = 1.4			
**229**	plakinamine N	NCI-60 screen = 11.5			
**230**	plakinamine O	NCI-60 screen = 2.4			

**Table 18:** Cytotoxic activity of manzamine alkaloids.

	**Compound**	**Cell Line (IC_50_ µM)**	**Source**	**Place of** **Collection**	**Ref.**
**231**	(+)-8-hydroxymanzamine A	SK-MEL *=* 0.83 KB = 1.38 BT-549 *=* 1.32 HepG2 = 0.90 LLC-PK11 = 2.21	*Acanthostrongylophora inges*	Papua New Guinea	[98]
**232**	(+)-8-manzamine A	SK-MEL = 1.82 KB = 1.82 BT-549 = 1.82 HepG2 = 8.02 LLC-PK11 = 3.92			
**231 a**	(+)-8-hydroxymanzamine A hydrochloride	SK-MEL = 1.30 KB = 1.15 BT-549 = 1.75 HepG2 = 2.5 LLC-PK11 = 3.08			
**232 a**	(+)-8 manzamine A hydrochloride	SK-MEL = 0.97 KB = 0.56 BT-549 = 1.50 HepG2 = 2.65 LLC-PK11 = 1.18			

**Table 19:** Cytotoxic activity of diterpen alkaloids.

	Compound	Cell Line (IC_50_ µM)	Source	Place of Collection	Ref.
**233**	agelasine E	CLL = 16	*Agelas citrine* and *Agelas nakamurai*	Caribbean Sea	[99,101]
**234**	19-oxofasciospongine A	LNCaP = 21.8 LU = 5 MCF-7 = 13.4	*Fasciospongia* sp.	Palau	[100]
**235**	iso-agelasine C	HL-60 = 25.3 K562 = 28.9 A549 > 50 HCT-116 = 38.8	*Agelas nakamurai*		[101]
**236**	agelasine J	HL-60 = 12.4 K562 = 16 A549 > 50 HCT-116 = 19.8			
**237**	nemoechine G	HL-60 = 18.4 K562 = 25.1 A549 > 50 HCT-116 = 33.9			

**Table 20:** Cytotoxic activity of sesquiterpene quinones/hydroquinones alkaloids.

	Compound	Cell Line (IC_50_ µM)	Source	Place of Collection	Ref.
**238**	(−)-4’-methylaminoavarone	HCT116 = 9 H4IIE = 40	*Dysidea avara*	Turkey	[102,103]
**239**	(−)-N-methylmelemeleone-A	HCT116 > 50 H4IIE > 50			
**240**	(−)-3’-methylaminoavarone	HCT116 = 45 H4IIE = 25			
